# Synthesis and Characterization of a 2,3-Dialkoxynaphthalene-Based Conjugated Copolymer via Direct Arylation Polymerization (DAP) for Organic Electronics

**DOI:** 10.3390/polym12061377

**Published:** 2020-06-19

**Authors:** Ignacio A. Jessop, Aylin Chong, Linda Graffo, María B. Camarada, Catalina Espinoza, Felipe A. Angel, Cesar Saldías, Alain Tundidor-Camba, Claudio A. Terraza

**Affiliations:** 1 Organic and Polymeric Materials Research Laboratory, Facultad de Ciencias, Universidad de Tarapacá, P.O. Box 7-D, Arica 1000007, Chile; aylinchong@gmail.com (A.C.); linda.graffo@gmail.com (L.G.); 2 Centro de Nanotecnología Aplicada, Facultad de Ciencias, Universidad Mayor, Santiago 8580745, Chile; maria.camarada@umayor.cl; 3 Núcleo de Química y Bioquímica, Facultad de Estudios Interdisciplinarios, Universidad Mayor, Santiago 8580745, Chile; 4 Departamento de Química Inorgánica, Facultad de Química y de Farmacia, Pontificia Universidad Católica de Chile, Santiago 7820436, Chile; cpespinoza@uc.cl (C.E.); faangel@uc.cl (F.A.A.); 5 Centro de Nanotecnología y Materiales Avanzados, CIEN-UC, Pontificia Universidad Católica de Chile, Santiago 7820436, Chile; 6 Departamento de Química Física, Facultad de Química y de Farmacia, Pontificia Universidad Católica de Chile, Santiago 7820436, Chile; casaldia@uc.cl; 7 Research Laboratory for Organic Polymers (RLOP), Facultad de Química y de Farmacia, Pontificia Universidad Católica de Chile, Santiago 7820436, Chile; atundido@uc.cl (A.T.-C.); cterraza@uc.cl (C.A.T.); 8 UC Energy Research Center, Pontificia Universidad Católica de Chile, Santiago 7820436, Chile

**Keywords:** dialkoxynaphthalene, benzothiadiazole, direct arylation polymerization, conjugated polymers, organic electronics

## Abstract

Poly[(5,5’-(2,3-bis(2-ethylhexyloxy)naphthalene-1,4-diyl)bis(thiophene-2,2′-diyl))-*alt*-(2,1,3-benzothiadiazole-4,7-diyl)] (PEHONDTBT) was synthesized for the first time and through direct arylation polymerization (DAP) for use as p-donor material in organic solar cells. Optimized reaction protocol leads to a donor-acceptor conjugated polymer in good yield, with less structural defects than its analog obtained from Suzuki polycondensation, and with similar or even higher molecular weight than other previously reported polymers based on the 2,3-dialkoxynaphthalene monomer. The batch-to-batch repeatability of the optimized DAP conditions for the synthesis of PEHONDTBT was proved, showing the robustness of the synthetic strategy. The structure of PEHONDTBT was corroborated by NMR, exhibiting good solubility in common organic solvents, good film-forming ability, and thermal stability. PEHONDTBT film presented an absorption band centered at 498 nm, a band gap of 2.15 eV, and HOMO and LUMO energy levels of −5.31 eV and −3.17 eV, respectively. Theoretical calculations were performed to understand the regioselectivity in the synthesis of PEHONDTBT and to rationalize its optoelectronic properties. Bilayer heterojunction organic photovoltaic devices with PEHONDTBT as the donor layer were fabricated to test their photovoltaic performance, affording low power-conversion efficiency in the preliminary studies.

## 1. Introduction

Conjugated polymers with alternating electron-donor and electron-acceptor units along their backbones (D−A CPs) have been investigated during the last decades for a variety of applications in organic electronics, such as organic photovoltaic (OPV) devices [1,2,3], organic light-emitting diodes (OLEDs) [4,5], organic field-effect transistors (OFETs) [6,7], polymer-based organic batteries [8,9], and chemical sensors [10,11]. CPs are typically prepared by versatile and reliable cross-coupling reactions, such as Suzuki–Miyaura, Migita–Stille, and Kumada–Corriu, among others, which tend to give high reactivity, high molecular-weight, and well-defined polymers. However, they require multiple synthetic steps, using expensive and toxic organometallic precursors. These disadvantages, including the residual impurities produced by organometallic compounds during synthesis, have a negative impact on the optoelectronic devices [12,13,14,15,16]. Palladium-catalyzed direct arylation polymerization (DAP), based on the C–H bond activation strategy, has recently emerged as a simple, eco-friendly, and long-term sustainable alternative to build carbon-carbon bonds between arenes [17,18,19,20,21]. However, DAP synthesis is prone to homo-coupling, branching, and cross-linking defects along the polymer backbones, mainly because of the lack of selectivity when the monomers have different aromatic C–H bonds, leading to poor performance in organic electronic applications, which limit their industrial application [22,23,24,25].

Dialkoxyphenylenes have been extensively studied as electron-donor building blocks in the synthesis of D–A CPs for optoelectronic applications [26,27,28]. Polymers based on dialkoxyphenylene moiety feature enhanced chain planarity, intermolecular ordering, and excellent thermal stability, while OPV devices involving this moiety as an active layer reached high conversion efficiency [26,27,28,29,30,31]. To fine-tune the optical and electrochemical properties of dialkoxyphenylene-based CPs, its replacement by dialkoxynaphthalene analogs with extended conjugation through their fused ring structure has been explored [32,33,34,35,36,37]. Among its structural isomers, the 2,3-dialkoxynaphthalene unit has been the least studied of the series, producing polymers with reduced yield and low molecular weight, which limits its field of applications [38,39]. These phenylene and naphthalene polymers are prepared by Suzuki or Stille couplings, but there are some recent examples of dialkoxyphenylene-based polymers successfully synthesized by DAP with high molecular weight [40,41,42,43]. These examples added to the significant advances made in preparing well-defined conjugated polymers via DAP [44,45,46,47,48,49,50,51,52], open the door to produce CPs based on 2,3-dialkoxynaphthalene. 

In this study, the DAP strategy was applied to synthesize and characterize the novel Poly[(5,5’-(2,3-bis(2-ethylhexyloxy)naphthalene-1,4-diyl)bis(thiophene-2,2′-diyl))-*alt*-(2,1,3-benzothiadiazole-4,7-diyl)] (PEHONDTBT) based on 2,3-bis(2-ethylhexyloxy)naphthalene (EHON) and 2,1,3-benzothiadiazole (BT), a well-known electron-acceptor building block for organic electronics [53,54,55,56]. Thiophene spacers (T) were inserted between EHON and BT to avoid the polymer backbone twisting by steric interactions between the monomers and to extend the electronic conjugation along the main chain. The optimized polymerization conditions: Pd_2_(dba)_3_ (2 mol%), P(*o*-OMePh)_3_ (8 mol%), Cs_2_CO_3_ (3 equiv), and PivOH (1 equiv) in toluene (0.5 M) for 3 h at 120 °C, gave PEHONDTBT with good yield, high molecular weight, and without structural defects in comparison with the same polymer prepared by Suzuki polycondensation reaction. Additionally, the batch-to-batch repeatability of the optimized DAP reaction was tested and demonstrated.

## 2. Materials and Methods 

### 2.1. Materials

All reagents, chemical compounds, and solvents were purchased from Sigma-Aldrich (Milwaukee, WI, USA), Merck (Darmstadt, Germany), and AK Scientific, Inc. (San Francisco) and used without any further purification. NBS was recrystallized twice from hot water, and THF was distilled from sodium/benzophenone ketyl under reduced pressure before use. The polymerization solvents were degassed and stored over activated 3 A° molecular sieves under an inert atmosphere, while the other solvents were ACS grade. 4,7-Dibromobenzo[*c*]-1,2,5-thiadiazole (BTBr) and 2,1,3-benzothiadiazole-4,7-bis(boronic acid pinacol ester) (BTBOR) were purchased from Sigma-Aldrich and used with no further purification. 4,7-Di(2-thienyl)-2,1,3-benzothiadiazole (BTDT) was prepared according to a procedure reported in the literature [31]. Synthesis of EHON derivatives are given in electronic supplementary information (ESI).

### 2.2. Measurements

^1^H and ^13^C NMR spectra were recorded on a Bruker AVANCE III HD 400 MHz spectrometer (Bruker Corporation, Karlsruhe, Germany) in deuterated solvents. Chemical shifts were reported as δ values (ppm) relative to tetramethylsilane (TMS). Size exclusion chromatography (SEC) was used to estimate the number-average (*M_n_*) and weight-average (*M_w_*) molecular weights of polymers, on a Wyatt Technology Dawn EOS HPLC (Wyatt Technology, Santa Barbara, CA, USA) instrument equipped with a Knauer pump, three PLgel 5µ Mixed-C columns, and a static light-scattering (EA-02 Dawn Eos Enhanced Optical System) detector. The flow rate was 1.0 mL·min^−1^ using tetrahydrofuran (THF) as eluent at 25 °C. All samples were prepared at 1.0 mg·mL^−1^ in THF and were filtered through a 0.45 µm nylon filter. Monodisperse polystyrene standards were used to prepare the calibration curve. Thermogravimetric analysis (TGA) and Differential scanning calorimetry (DSC) measurements were carried out with a Mettler Toledo TGA/STDA 851 and Mettler Toledo DSC821 (Mettler Toledo, Greifensee, Switzerland), respectively, under nitrogen atmosphere. TGA thermograms were taken at a heating rate of 10 °C·min^−1^, while DSC experiments were performed at heating and cooling rates of 10 °C·min^−1^ and 20 °C·min^−1^, respectively. UV-vis absorption spectra were recorded on a Shimadzu UV-1800 spectrophotometer (Shimadzu Corporation, Kyoto, Japan) using 1 cm path length quartz cells. For solid-state measurements, polymer solutions were spin-coated onto glass plates. Cyclic voltammetry (CV) was performed with a Voltalab PGZ100 potentiostat (Radiometer Analytical, Villeurbanne, France) using a platinum disk as a working electrode at a scan rate of 50 mV·s^−1^, and an Ag/AgCl as reference electrode (0.01 M AgNO_3_ in acetonitrile). The working electrode was modified by drop-casting 1% (*w*/*v*) solutions in chloroform. The electrolytic solution (0.1 M tetrabutylammonium tetrafluoroborate (Bu_4_NBF_4_) in acetonitrile) was purged with high purity argon for 15 min prior to each experiment, and a lower flow was maintained during the measurements. The oxidation potential (*E_ox_*) of ferrocene in these conditions was 0.315 V versus Ag/Ag^+^ and 0.447 V versus the saturated calomel electrode (SCE). The HOMO and LUMO energy levels were determined from the oxidation and reduction onset from the voltammogram profiles. The onsets were defined at the position where the current starts to differ from the baseline. As reported in literature, the absolute potential of the SCE electrode is -4.7 eV in vacuum [57].

### 2.3. Theoretical Calculations

**Scheme 1:**
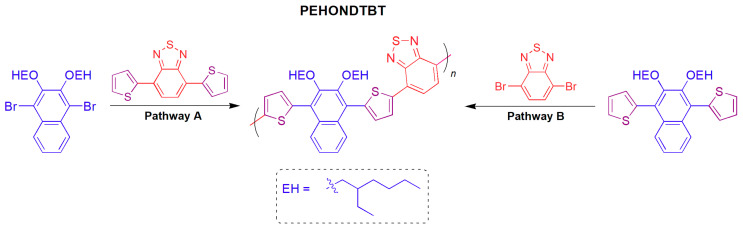
Synthetic pathways for obtaining PEHONDTBT via DAP.

The geometry of the PEHONDTBT and the monomers of reaction pathways A and B (see Scheme 1), were fully optimized at the density functional theory (DFT) level using the Gaussian16 computational package [58], implementing the Becke’s three parameters nonlocal hybrid exchange potential with the nonlocal correlation functional of Lee, Yang, and Parr (B3LYP) without any symmetry restriction [59,60,61], with a triple-ζ basis set TZVP for all atoms [62]. Each of the dihedral angles between the aromatic rings was scanned by internal rotations to explore the potential energy surface and find the lowest energy conformation. Dimer and trimer conformations were optimized at the same level of theory to compare the effect of the chain length on the frontier orbitals and geometry. 

The (P(CH_3_)_3_)Pd(Ph)(CH_3_COO^−^) model was used as a platform to test the activation barrier of the different C–H bonds of each substrate during the concerted-metalation deprotonation (CMD) transition state. This method has been successfully applied by Gorelski and coworkers to explain and predict the regioselectivity of palladium-catalyzed direct arylation [63]. The activation energy (*E_a_*) resulting from this protocol refers to the Gibbs free energy of the transition state of the CMD, referenced to the substrate and model catalyst (ΔG^‡^_298_). A triple-ζ basis set TZVP was used for all atoms [62], excepting for Pd atoms, where the DZVP basis set was applied [64]. In pathway B, the alkyl chain –EH, was reduced to –CH_3_ to lower the computational costs. The transition states were located and confirmed by frequency calculations (single imaginary frequency). From these calculations, it is also possible to estimate the selectivity ratio between H_α_ and H_β_ at the temperature of polymerization by Arrhenius’s law. All the transition state geometries are summarized in ESI.

### 2.4. OPV Devices Fabrication

1.5 × 1.5 inch glass substrates with a 1000 Å pre-patterned indium tin oxide (ITO) layer (Tinwell Technology Ltd., Hong Kong, China) were used for device fabrication. The substrates exhibited ~15 Ω·sq^−1^ surface resistance and ~90% optical transparency over the solar spectrum. The cleaning procedure of the substrates, conducted inside a laminar flow hood, included scrubbing with detergent solution, rinsing with DI water, and ultrasonic baths for 10 min of DI water, acetone, and isopropanol. Lastly, nitrogen gas was used to dry the substrates. All films based on small-molecules were prepared by vapor deposition in a vacuum chamber (< 5.0·10^−6^ Torr) with a 1000 Å top aluminum electrode, at a constant rate of 5.0 Å·s^−1^ for aluminum. The layer sequence is: A 10 nm layer of molybdenum oxide (MoO_x_) was deposited at about 0.3 Å·s^−1^ on ITO as the hole-injecting layer (HIL). The B3a polymer was dissolved in chlorobenzene at a concentration of 8 mg·mL^−1^, 10 mg·mL^−1^, 12 mg·mL^−1^, 15 mg·mL^−1^, 18 mg·mL^-1^, and 20 mg·mL^−1^. The organic solutions were deposited by spin-coating, using 100 µL of the solution on each substrate (ITO|MoO_x_), at 2500 rpm for 10 s and then 3500 rpm for 40 s. The devices were completed with vacuum deposition of a 40 nm layer of fullerene (C_60_) as the donor layer, 8 nm of 4,7-diphenyl-1,10-phenanthroline (Bphen) as the electron transport layer (ETL), and 2 nm of 8-hydroxyquinolinato lithium (Liq) as the electron-injecting layer (EIL). The deposition rates for C_60_ and Bphen were 4.0 Å·s^−1^, and for Liq was 1.0·Å s^−1^. Four identical OPV devices with an active area of 0.1 cm^2^ were produced on a single substrate. The thickness of the films was measured with a Profilm3D optical profilometer (Filmetrics, Unterhaching, Germany). The current density–voltage (JV) characteristics were recorded with a Keithley 2400 source meter (Keithley Instruments, Inc., Cleveland, Ohio, USA) in the dark and under irradiation at 80 mW·cm^−2^ generated by a Solux 3SS4736 with a 50W 47K halogen lamp. The intensity was calibrated with a Hamamatsu S1787-12 silicon photodiode. External quantum efficiency (EQE) measurements were performed with a SpectraPro 275 monochromator (Action Research Corp., Acton, Massachusetts, USA). 

### 2.5. General Procedure for the Synthesis of PEHONDTBT *via* DAP

A 10 mL oven-dry microwave vial was charged with 1 equivalent (eq) of each monomer EHON and BTDz, a mol% amount of catalyst, a mol% amount of ligand P(*o*-OMePh)_3_ (1:4 ligand/catalyst), 3 eq of Cs_2_CO_3_, and 1 eq of pivalic acid (PivOH). The vial was sealed and purged with vacuum and nitrogen filling (3 cycles). Anhydrous and oxygen-free solvent was then added (0.4 mol·L^−1^ or 0.5 mol·L^−1^), and the mixture was stirred at room temperature for 15 min to afford the dissolution of the monomers. The vial was placed in a pre-heated oil bath at 120 °C until the gelation of the reaction mixture. The reaction was cooled to 90 °C, and 2.0 mL of chlorobenzene (CB) was added, stirring the reaction mixture for an additional 5 min. The mixture was cooled to room temperature and poured in a 9:1 *v*/*v* methanol/acidified water (10% *v*/*v* HCl) solution. The precipitated was filtered through a Soxhlet thimble and washed using a Soxhlet apparatus with acetone and *n*-hexane. The polymer was extracted with chloroform, and the solution was concentrated to 5–10 mL and poured into methanol. The precipitated was filtered through a 0.45 µm nylon filter and vacuum-dried to afford a red solid. 

## 3. Results and Discussion

### 3.1. Synthesis and Characterization of PEHONDTBT

As stated by Livi et al. for a 1,4-dialkoxyphenylene-based polymer [43], there are two pathways to synthesize PEHONDTBT (Scheme 1). In pathway A, the thiophene spacers are flanking the EHON unit, while in pathway B, the thiophenes are coupled to the 2,1,3-benzothiadiazole moiety. We tested both paths using the following synthetic conditions: equimolar amount of monomers (0.4 or 0.5 mol·L^−1^), catalyst (2 or 5 mol%), P(*o*-OMePh)_3_ (1:4 catalyst/ligand), Cs_2_CO_3_ (3 equiv), and PivOH (1 equiv) in THF or toluene at 120 °C. The reaction was stopped upon gelation of the reaction mixture. After precipitation in methanol, the polymers were purified by successive Soxhlet extractions using acetone, *n*-hexane, and chloroform. The polymer was soluble in chloroform, THF, and chlorobenzene at room temperature, mainly because of the branched 2-ethylhexyloxy groups in the naphthalene ring.

To optimize the DAP reaction, conditions such as solvent, catalyst, and concentration of the species were examined. The results are summarized in Table 1. First, the system Pd_2_dba_3_•CHCl_3_/THF for pathway A (entry A1) was tested. Successful conditions reported by Roy et al. and Morin et al. for the synthesis of a 1,4-dialkoxyphenylene- and a BTDT-based D-A CPs [42,51], were ineffective to produce PEHONDTBT. Then, the catalyst was replaced with Pd(OAc)_2_ and increased its concentration (2 mol% to 5 mol%), which resulted in the polymer with the highest number-average molecular weight of the series after 2 h of reaction, but in low yield (entry A2, 20% yield and *M_n_* = 20 kDa). Two more solvents, DMAc (entry A3) and toluene (entry A4), a more polar and a less polar solvent than THF, respectively, were examined and the reaction yield improved with the latter (entry A4. 54% yield and *M_n_* = 2.5 kDa), but the number-average molecular weight was lower and the reaction time was longer than entry A2. Surprisingly, the catalyst Pd(OAc)_2_ showed higher reactivity in a low polar solvent than in a highly polar solvent, which is contradictory to previous reports [14,18]. The moderate yield and number-average molecular weight of PEHONDTBT synthesized through pathway A could be attributed to the steric hindrance exerted by the alkoxy chains adjacent to the C–Br bonds in the EHON monomer and the bulkiness of the catalytic system, which could have hindered the interactions between EHON and the catalyst during the reaction. A more detailed analysis will be performed in Section 3.2.

Alternatively, the study of pathway B with the systems Pd(OAc)_2_/THF and Pd_2_(dba)_3_•CHCl_3_/THF (entries B1 and B2) was conducted. The conditions used in the entry B2 produced a polymer in higher yield and number-average molecular weight compared to entry A1 (entry B2, 33% yield, and *M_n_* = 24 kDa). Note that in all entries where THF was the reaction solvent, a large amount of insoluble material remained in the Soxhlet thimble. These residues were insoluble even in hot chlorobenzene, *o*-dichlorobenzene, or 1,2,4-trichlorobenzene, which is indicative of the formation of very high molecular weight polymers and/or with structural defects. To improve the reaction yield, the system Pd_2_(dba)_3_•CHCl_3_/toluene was evaluated. A significant increase in yield and molecular weight was accomplished with this system (entry B3a, 72% yield, and *M_n_* = 30 kDa). These results clearly show the effect of solvent polarity to obtain polymers in high yields and with high molecular weights by DAP. Other catalysts (entries B4 and B5) and the catalyst/ligand ratio (entries B6 y B7) were also tested, with no better results than those obtained for B3a. 

In comparison with other 2,3-dialkoxynaphthalene-based polymers previously reported and synthesized through cross-coupling reactions [36,37], PEHONDTBT obtained by DAP exhibited similar or even higher molecular weight. PEHONDTBT was also synthesized via Suzuki polycondensation reaction (SPC, entry S1), exhibiting lower yield, and molecular weight than B3a. Contrary to Livi et al. [43], our results show that pathway B is more efficient than pathway A to synthesize dialkoxynaphthalene-based CPs. The literature on BT-based polymers synthesized through DAP supports our results [13,55,56], where the use of the BT monomer as the dibromo derivative leads to polymers with higher molecular weights and less structural defects. The theoretical calculations in Section 3.2. provide insight to understand the difference between the pathways better.

**Figure 1:**
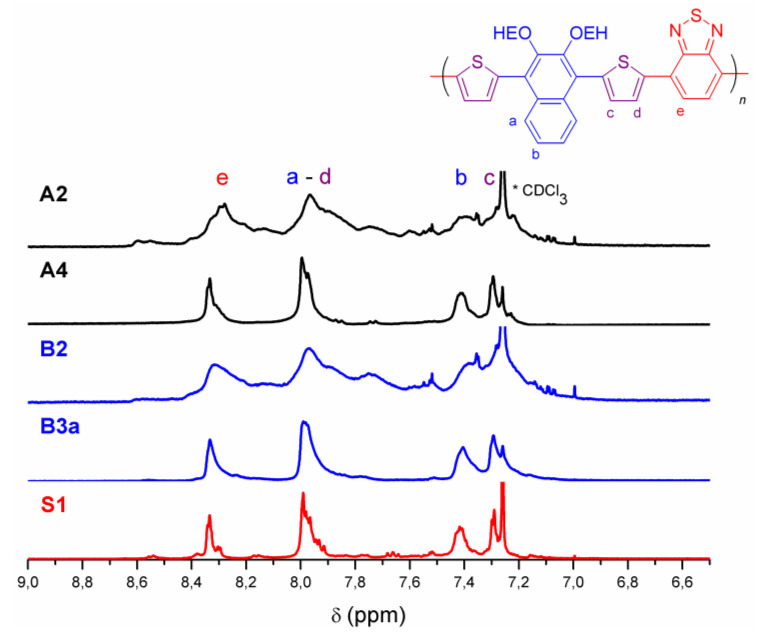
^1^H NMR spectra of the aromatic region of entries A2, A4, B2, B3a, and S1 in CDCl_3_.

All polymers were characterized by ^1^H NMR analyses (Figures S1–S12). Although insoluble materials were left in the Soxhlet cartridge, the chloroform-soluble fractions of all entries revealed practically the same spectra compared to entry S1 (DAPs and Suzuki). The main signals in the spectra at 8.3, 8.0, 7.4, and 7.3 ppm are attributable to the hydrogen atoms on the BT, EHON, and T rings, while the small signals along are attributable to end-groups or structural defects such as homo-couplings, branching, and/or cross-linking (Figure 1). As seen in Figure 1, polymers synthesized in toluene (entries A4 and B3a) have cleaner and narrower aromatic signals than THF (entries A2 and B2). While the polymer obtained by Suzuki reaction (S1) exhibit signals of structural defects, only the main peaks are observed for B3a, which indicates its well-defined structure.

To examine the reproducibility of the synthetic strategy, two more samples were prepared using the same conditions of entry B3a (entries B3b and B3c). ^1^H NMR spectra show that B3b and B3c are practically identical to B3a (Figures S5–S7), although their molecular weights are relatively higher than B3a (Table 1). Their thermal, optical, and electrochemical properties are summarized in Table 2. Thermal properties were tested by TGA and DSC. The polymers exhibited good thermal stability with 5% weight loss at a temperature higher than 380 °C (Figure S13). The DSC curves show that B3a-c displays thermal transitions in the second heating cycle, which could be attributable to a reorganization process of the polymeric chains (Figure S14).

**Figure 2:**
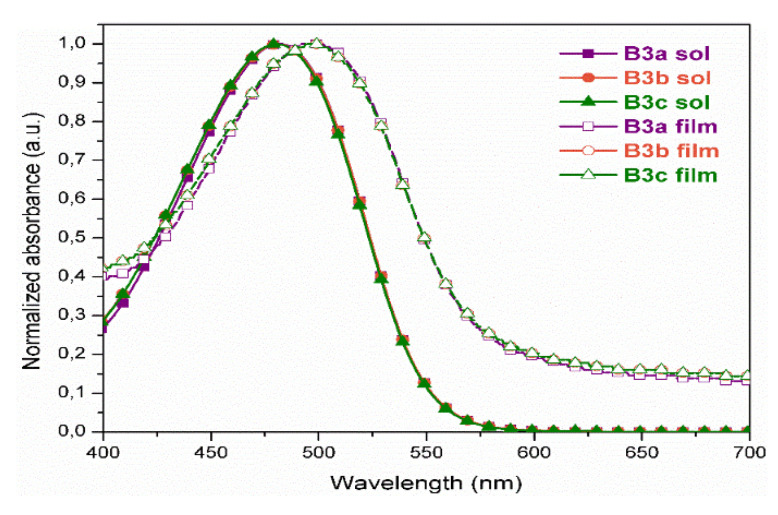
UV-vis spectra of B3a-c in CHCl_3_ dilute solution and thin films.

Absorption spectra of polymers in solution synthesized under different reaction conditions were recorded to compare their optical properties. The polymers exhibit two distinctive bands resulting from the π–π* transition, centered at about 330 nm, and the intermolecular charge-transfer (ITC), at about 480 nm (Figure S15). As seen in Table 1, polymers synthesized in THF showed red-shifted maximum absorption peaks (488–489 nm) in comparison with those prepared in toluene (480–484 nm). This could be due to longer average conjugation lengths, which depend on the maximum effective conjugation length, degree of polymerization, and structural defects [65]. The optical properties of B3a-c were also studied in spin-coated thin films (Figure 2). As expected, the ITC peaks are red-shifted compared with those in solution for the agglomeration and enhanced π–π interactions between polymer chains in the solid-state [66,67]. The B3a-c polymers exhibit identical optical band gap values in chloroform solution and thin-films, 2.24 and 2.15 eV, respectively, calculated from the absorption onsets. In comparison with previously reported 2,6-dialkoxynapthalene- (*λ_max_* = 552 nm, *E_g_^opt^* = 1.86 eV) and 1,4-dialkoxyphenylene- (*λ_max_* = 603 nm, *E_g_^opt^* = 1.70 eV) based CPs [31,33], a clear hypsochromic-shift in absorption maxima of the thin-film was observed for PEHONDTBT (*λ_max_* = 498 nm, *E_g_^opt^* = 2.15 eV, Table 2), increasing its band gap value. The short conjugation length along the polymer backbone caused by its twisted conformation, explains this phenomenon (see Section 3.2 for details). 

HOMO energy levels of B3a-c were calculated from the oxidation onsets obtained from cyclic voltammetry (Table 2 and Figure S16), while the LUMO levels were estimated from *E_g_^opt^* film and the HOMO levels, as the reduction potential waves were poorly resolved. The three polymers exhibit similar features, with the HOMO at about −5.3 eV and the LUMO at about −3.2 eV. According to the literature [30,43], 1,4-dialkoxyphenylene-based CPs exhibit their HOMO level at about −5.44 eV. The higher HOMO value of PEHONDTBT compared to 1,4-dialkoxyphenylene-based CPs could be attributed to its less planar and conjugated structure.

In summary, the thermal, optical, and electrochemical properties of the three polymers B3a-c are practically the same. These values, along with the yields and molecular weights obtained, show that the DAP reaction is an efficient and batch-to-batch repeatable strategy for the synthesis of well-defined PEHONDTBT.

### 3.2. Theoretical Calculations

**Figure 3:**
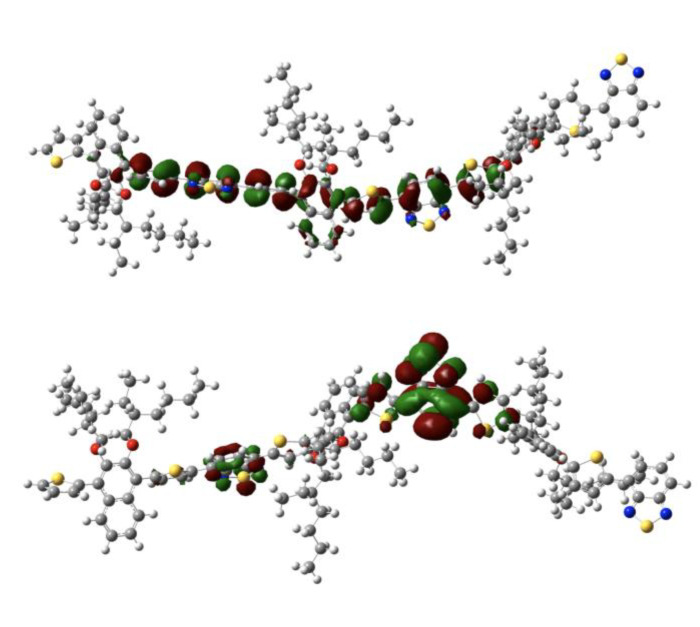
Isosurfaces of the HOMO (up) and LUMO (down) frontier orbitals of the PEHONDTBT trimer at B3LYP/TZVP level.

To better understand and complement the experimental data, DFT calculations of polymers and monomers were carried out. First, the ground state geometry of PEHONDTBT monomer was optimized by performing the internal rotation of the dihedral angles linking the thiophene units to the central naphthalene ring, at B3LYP/TZVP level of theory. Subsequently, the dimer and the trimer structures were also optimized at the same level. The vibrational analysis confirmed the optimized geometries as local minima. The trimer was selected as a representation of the polymer. Figure 3 shows a plot of the frontier orbitals of the trimer, mainly centered in the benzothiadiazole moieties in the LUMO’s case. The calculated band gap is 2.58 eV (E_HOMO_ = −5.49 eV and E_LUMO_ = −2.91 eV). Figure S17 summarizes the dihedral angles between the naphthalene and thiophene ring, as the chain grows. As seen, the optimized geometry of the trimer presents a twisted conformation, which has a direct impact on the stability of the polymer and the delocalization of the electronic density, explaining its poorer electro-optical properties in comparison with D–A CPs based on DTBT and 1,4-dialkoxyphenylene or 2,6-dialkoxy naphthalene.

**Figure 4:**
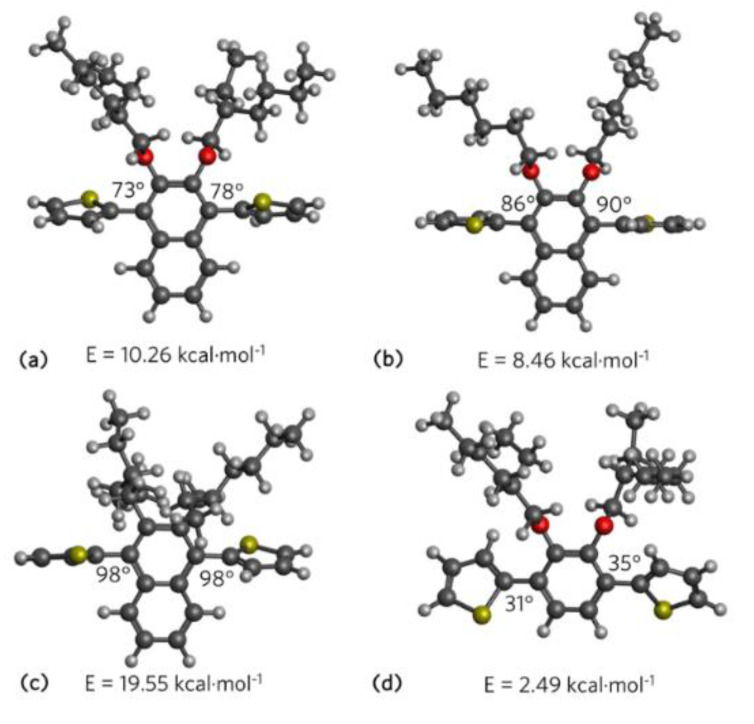
Dihedral angles of (**a**) EHON, (**b**) the monomer with *n*-hexyloxy side chains, (**c**) the monomer with *n*-ethylhexyl- side chains, and without the oxygen atoms, and (**d**) 2,3-dialkoxyphenylene. The energetic barriers (E in kcal·mol^−1^) are informed for the rotation of the thiophene rings.

To understand the origin of the nonplanarity along with the PEHONDTBT structure, different monomers were designed, excluding the benzothiadiazole moiety: EHON (Figure 4a), the monomer with *n*-hexyloxy side chains (Figure 4b), the monomer with *n*-ethylhexyl- side chains and without the oxygen atoms (Figure 4c) and finally, the 2,3-dialkoxyphenylene ring (Figure 4d). Dihedral angles of the ground-state conformations and the rotational barrier of the second thiophene ring are displayed in Figure 4. The 2,3-dialkoxyphenylene monomer presented the lowest rotational barrier and higher planarity. This result was expected, based on several optoelectronic applications that exploit the nearly-planar conformation between dialkoxyphenylene and thiophene [30]. These results show that the conjugation lost in the PEHONDTBT is mainly due to the naphthalene ring, whose hydrogen atoms at the 5,8-positions prevent and hinder the co-planarization with the thiophene-flanking rings. The results support what was previously stated by Samanta et al., who hypothesize that the large bandgap of 1,4-polynaphtalene is caused by the high torsion angle between monomers due to the peri-hydrogens in the naphthalene units [38].

**Figure 5:**
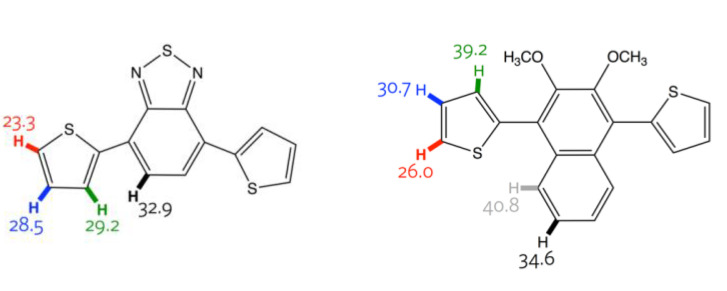
Gibbs free energy of the concerted-metalation deprotonation (CMD) transition state associated with the activation energy of the hydrogen atoms. All calculations were performed at DFT level.

As previously mentioned, the polymerization through pathway B produced high yield and purity polymers, while pathway A was less efficient. DFT calculations can estimate the activation energy of each C–H bond available on the monomers, which is useful information to predict the regioselectivity of the DAP reaction [22]. As shown in Figure 5, for monomer A belonging to pathway A, the difference in the activation energy between the α and β protons is 5.2 kcal·mol^−1^. This value is close to the information reported by Roy et al. (6.0 kcal·mol^−1^), calculated with a previous version of the computational program used in the present study [42]. The Arrhenius’s law allows calculating the selectivity of the α-position at the temperature of polymerization (120 °C). For pathway A, a ratio of approximately 800/1 favors the H_α_ polymerization over the H_β_. In the case of pathway B, the ethylhexyloxy chains on EHON were replaced by methyl groups to decrease the computational costs. The difference in the activation energy between the H_α_ and H_β_ is 4.7 kcal·mol^−1^, while the selectivity ratio is approx. 450/1, which explains the well-defined structure of PEHONDTBT.

Although these results show a higher selectivity and lower activation energy in pathway A, they do not explain the experimental evidence correctly. In this sense, it is necessary to consider the spatial conformation of the monomers when they are close enough to react. As Figure S18 displays, in pathway A the alkoxy chains might hinder the attachment of the activated monomer to the aryl derivative. The cavity space between the alkoxy chain and the naphthalene ring is about 3 Å. The thiophene ring must enter perpendicularly to the naphthalene to avoid repulsive interactions. This also supports the hypothesis of Samanta et al., that justifies the low molecular weight obtained for its 1,4-polynapthalene due to the steric hindrance exerted by the side chains close to the reaction center in the naphthalene monomer [38]. In pathway B, the thiophene is already attached to the naphthalene, acting as a spacer, being more available for the polymerization. 

### 3.3. Photovoltaic Characteristics

**Figure 6:**
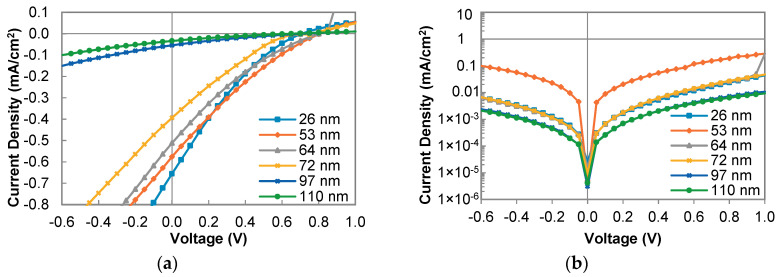
(**a**) Light density–voltage (JV) characteristics and (**b**) dark JV characteristics for bilayer organic photovoltaic (OPV) devices.

**Figure 7:**
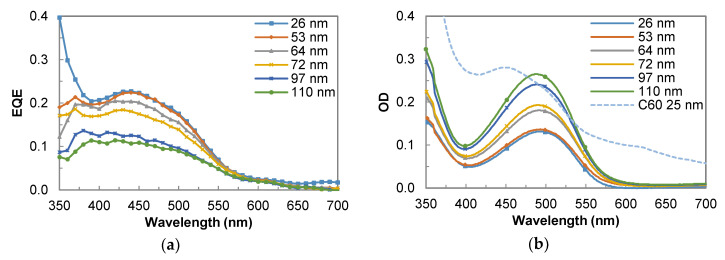
(**a**) External quantum efficiency (EQE) for bilayer OPV devices and (**b**) thickness dependent absorption spectra of PEHONDTBT films and 25 nm layer of C_60_.

To test the photovoltaic properties of PEHONDTBT, a bilayer heterojunction OPV device with B3a as the donor and C_60_ as the acceptor was the basis of this study. The layer structure is the following: ITO|MoO_x_(10 nm)|PEHONDTBT(26–110 nm)|C_60_(40 nm)|Bphen(8 nm)|Liq(2 nm)|Al(100 nm), which includes MoO_x_ as the hole-injection layer, Bphen as the electron-transport layer, and Liq as the electron-injection layer. The thickness of the B3a layer was modified by spin-coating different concentrations of B3a solutions in chlorobenzene: 8, 10, 12, 15, 18, and 20 mg·mL^−1^, obtaining film thickness values of 26, 53, 64, 72, 97, and 110 nm, respectively. Light and dark JV characteristics are shown in Figure 6a,b (device data is summarized in Table S1). The short-circuit current (*J_sc_*) for bilayer heterojunction OPV devices is limited by the thickness of the active layer. Exciton diffusion length (*L_D_*) for organic materials, particularly polymers, are typical ~ 10 nm [68]. Therefore, only thickness values close to L_D_ effectively generate photocurrent. Beyond this value, excitons generated by the bulk film cannot diffuse to the donor-acceptor interface for charge generation, filtering out photons absorbed at the interface. The film thicknesses produce *J_sc_* for values ranging from 0.66 mA·cm^−2^ to 0.03 mA·cm^−2^. This thickness dependence can be observed in the dark JV characteristics as well, where all devices have a diode behavior with ohmic conduction regimes. Likewise, the thickness dependence is observed in the spectral response measurements, as shown in Figure 7a. The spectral response for the 26 nm device matches quite well the absorption spectra of the donor and acceptor materials, with a peak at ~450 nm, corresponding to the contribution of C_60_, and a second peak at ~500 nm, corresponding to the polymer (Figure 7b). As the film thickness increases, as expected, the photocurrent efficiency is reduced throughout the entire spectra. Particularly, at short wavelength regions, where the polymer has high optical density. Regardless of the low efficiencies obtained, the polymer can be used as a donor material in bilayer heterojunction OPV devices. These results are preliminary, as bulk heterojunction OPV devices will follow this work.

## 4. Conclusions

In this study, the synthesis and characterization of the novel D–A CP PEHONDTBT through DAP was presented. The optimized conditions allowed to obtain PEHONDTBT in good yield (> 70%), with similar or even higher molecular weight (*M_n_* = 30 kDa) than some previously reported 2,3-dialkoxynaphthalene-based CPs, and with less structural defects than the analog synthesized via Suzuki polycondensation protocol (S1, *M_n_* = 6.0 kDa). The batch-to-batch repeatability of the DAP reaction for the synthesis of PEHONDTBT was also demonstrated in this work. PEHONDTBT exhibited good solubility in a variety of organic solvents and thermal stability (TDT_5%_ > 380 °C). Due to its twisted conformation, mainly attributed to hindered orbital overlap between the naphthalene and thiophene rings, PEHONDTBT has a blue-shifted absorption maximum and a higher optical band gap than D–A CPs based on DTBT and 1,4-dialkoxyphenylene or 2,6-dialkoxy naphthalene. Preliminary photovoltaic studies based on PEHONDTBT were carried out to demonstrate that polymer can be used as the p-donor layer in organic electronic devices.

## Figures and Tables

**Table 1:** Optimization of the DAP reaction for the synthesis of PEHONDTBT.

Entry	Catalyst (%)	Solvent	Time (h)	Yield (%) ^3^	*M_w_* (kDa)	*M_n_* (kDa)	PDI	λ_max_^abs^ (nm) ^6^
**A1**	Pd_2_(dba)_3_•CHCl_3_ (2)	THF	0.5	- ^4^	-	-	−	-
**A2**	Pd(OAc)_2_ (5)	THF	2.0	20 ^5^	37	20	1.9	489
**A3**	Pd(OAc)_2_ (5)	DMAc	24	- ^4^	-	-	-	-
**A4**	Pd(OAc)_2_ (5)	Toluene	21	54	4.1	2.5	1.6	484
**B1**	Pd(OAc)_2_ (5)	THF	1.5	13 ^5^	110	19	5.8	488
**B2**	Pd_2_(dba)_3_•CHCl_3_ (2)	THF	2.0	33 ^5^	83	24	3.5	488
**B3a**	Pd_2_(dba)_3_•CHCl_3_ (2)	Toluene	3.0	72	72	30	2.4	482
**B3b**	Pd_2_(dba)_3_•CHCl_3_ (2)	Toluene	3.0	79	127	49	2.6	481
**B3c**	Pd_2_(dba)_3_•CHCl_3_ (2)	Toluene	5.0	74	105	43	2.4	480
**B4**	Pd(Herrmann) (2)	Toluene	5.0	69	78	20	3.9	482
**B5**	Pd(OAc)_2_ (2)	Toluene	3.0	64	42	18	2.3	482
**B6** ^1^	Pd_2_(dba)_3_•CHCl_3_ (2)	Toluene	5.0	67	67	34	2.0	482
**B7** ^2^	Pd_2_(dba)_3_•CHCl_3_ (2)	Toluene	5.0	38	19	14	1.4	481
**S1** **(SPC)**	Pd[PPh_3_]_4_ (4)	Toluene	48	18	9.0	6.0	1.5	478

^1^ 1:2 Pd/L ratio. ^2^ 1:8 Pd/L ratio. ^3^ From chloroform fraction. ^4^ All reaction mixture was recovered from the acetone fraction. ^5^ Insoluble material was left in the Soxhlet thimble after extraction with chloroform. ^6^ In chloroform dilute solution.

**Table 2:** Thermal, optical, and electrochemical properties of PEHONDTBT.

Entry	TDT_5%_ (°C) ^1^	*λ_max_^sol^* (nm) ^2^	*E_g_^opt^* sol (eV) ^3^	*λ_max_^film^* (nm) ^2^	*E_g_^opt^* Film (eV) ^3^	E_HOMO_ (eV) ^4^	E_LUMO_ (eV) ^5^
**B3a**	381	482	2.24	498	2.15	−5.31	−3.17
**B3b**	381	482	2.24	496	2.15	−5.35	−3.20
**B3c**	381	482	2.24	496	2.15	−5.47	−3.32

^1^ Temperature at 5% weight loss. ^2^ Absorption maximum taken from UV-Vis spectra of the polymer in chloroform dilute solution or in solid-state. ^3^ Calculated from the absorption edge of the polymer in solution or in thin-film: *E_g_^opt^* = 1240·*λ_edge_*^−1^ eV. ^4^ E_HOMO_ = −e(*E_on_^ox^* + 4.4) eV. ^5^ E_LUMO_ = E_HOMO_ + *E_g_^opt^* film.

## Supplementary Materials

The following are available online at https://www.mdpi.com/2073-4360/12/6/1377/s1, Synthesis and characterization of EHON derivatives, Scheme S1. Synthetic route to EHON derivatives, Synthesis of polymers, Figure S1. ^1^H NMR spectra of A2 entry in CDCl_3_, Figure S2. ^1^H NMR spectra of A4 entry in CDCl_3_, Figure S3. ^1^H NMR spectra of B1 entry in CDCl_3_, Figure S4. ^1^H NMR spectra of B2 entry in CDCl_3_, Figure S5. ^1^H NMR spectra of B3a entry in CDCl_3_, Figure S6. ^1^H NMR spectra of B3b entry in CDCl_3_, Figure S7. ^1^H NMR spectra of B3c entry in CDCl_3_, Figure S8. ^1^H NMR spectra of B4 entry in CDCl_3_, Figure S9. ^1^H NMR spectra of B5 entry in CDCl_3_, Figure S10. ^1^H NMR spectra of B6 entry in CDCl_3_, Figure S11. ^1^H NMR spectra of B7 entry in CDCl_3_, Figure S12. ^1^H NMR spectra of S1 entry in CDCl_3_, Thermal characterization, Figure S13. TGA thermograms of B3a–c, Figure S14. DSC traces for second heating for B3a–c under nitrogen at speed scanning of 10 °C·min−^1^, UV-vis characterization, Figure S15. UV-vis of all polymers synthesized in chloroform dilution solution, Electrochemical characterization, Figure S16. Cyclic voltammograms of (a) B3a, (b) B3b, and (c) B3c. Scan rate = 50 m·Vs^−1^, Theoretical calculations, Figure S17. Dihedral angles of the monomer, dimer, and trimer of PEHONDTBT, determined from theoretical simulations. All calculations were performed at B3LYP/TZVP level, Figure S18. Scheme of the polymerization pathways, highlighting the reduced cavity available for the coupling between the BTDT and the aryl bromide derivative (EHONBr), Cartesian coordinates for the calculated species, Photovoltaic characterization, Table S1. Photovoltaic characteristics for OPV devices.
