# Supplementary material for: Synthesis and Characterization of a 2,3-Dialkoxynaphthalene-Based Conjugated Copolymer via Direct Arylation Polymerization (DAP) for Organic Electronics
Source: Polymers (Basel). 2020 Jun 19;12(6):1377. doi: 10.3390/polym12061377 (PMC7362231; doi:10.3390/polym12061377)
Supplement: Supplementary file 1 [file polymers-12-01377-s001.pdf]

polymers
MDPI

Electronic Supplementary Information - ESI

Synthesis and Characterization of a 2,3-Dialkoxynaphthalene-based Conjugated Copolymer via Direct Arylation Polymerization (DAP) for Organic electronics

Ignacio A. Jessop[1*], Aylin Chong[1], Linda Graffo[1], María B. Camarada[2,3], Catalina Espinoza[4], Felipe A. Angel[4,5], Cesar Saldías[6], Alain Tundidor-Camba[7,8] and Claudio A. Terraza[7,8]

1 Organic and Polymeric Materials Research Laboratory, Facultad de Ciencias, Universidad de Tarapacá. P.O. Box 7-D, Arica 1000007, Chile; iajessop@uta.cl (I.A.J.); aylinchong@gmail.com (A.C.); linda.graffo@gmail.com (L.G.)
2 Centro de Nanotecnología Aplicada, Facultad de Ciencias, Universidad Mayor, Santiago 8580745, Chile; maria.camarada@umayor.cl (M.B.C.)
3 Núcleo de Química y Bioquímica, Facultad de Estudios Interdisciplinarios, Universidad Mayor, Santiago 8580745, Chile; maria.camarada@umayor.cl (M.B.C.)
4 Departamento de Química Inorgánica, Facultad de Química y de Farmacia, Pontificia Universidad Católica de Chile, Santiago 7820436, Chile; cpespinoza@uc.cl (C.E.); faangel@uc.cl (F.A.A.)
5 Centro de Nanotecnología y Materiales Avanzados, CIEN-UC, Pontificia Universidad Católica de Chile, Santiago 7820436, Chile; faangel@uc.cl (F.A.A.)
6 Departamento de Química Física, Facultad de Química y de Farmacia, Pontificia Universidad Católica de Chile, Santiago 7820436, Chile; casaldia@uc.cl (C.S.)
7 Research Laboratory for Organic Polymers (RLOP), Facultad de Química y de Farmacia, Pontificia Universidad Católica de Chile, Santiago 7820436, Chile; atundido@uc.cl (A.T.-C.); cterraza@uc.cl (C.A.T.)
8 UC Energy Research Center, Pontificia Universidad Católica de Chile, Santiago 7820436, Chile; atundido@uc.cl (A.T.-C.); cterraza@uc.cl (C.A.T.)
* Correspondence: iajessop@uta.cl

Synthesis and characterization of EHON derivatives

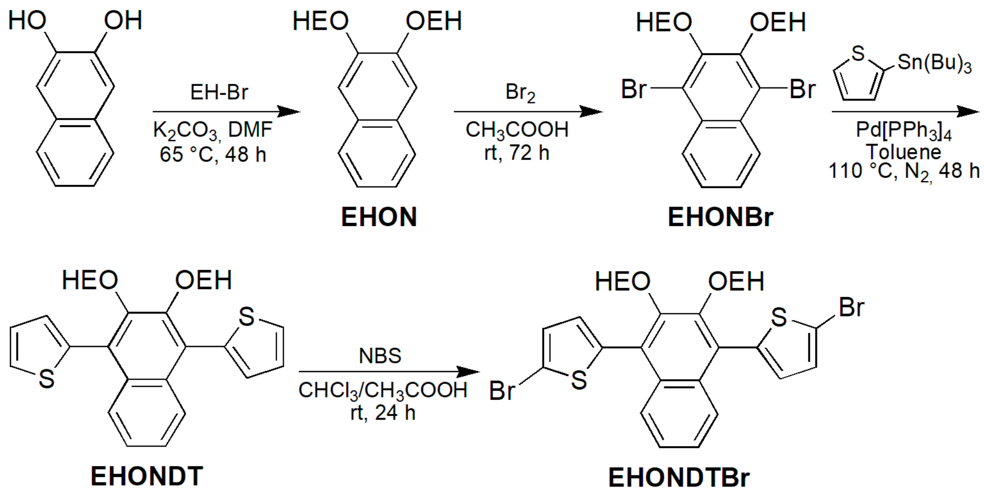

Scheme S1. Synthetic route to EHON derivatives.

2,3-Bis(2-ethylhexyloxy)naphthalene (EHON). A mixture of 2,3-dihydroxynaphthalene (1.00 g, 6.24 mmol), K_2CO_3 (8.62 g, 62.4 mmol) and DMF (30 mL) was stirred at 65 °C for 30 min. Then, 2-ethylhexyl bromide (EH–Br) (3.61 g, 18.7 mmol) was added dropwise and the reaction mixture was stirred for 48 h at 65 °C. After cooling to room temperature, water was added and the reaction mixture was extracted with chloroform. The combined organic layers were dried over anhydrous Na_2SO_4, filtered and the solvent was removed under vacuum affording an orange oil, which was used directly in the next step without further purification (2.30 g, 96 %). ^{1}H NMR ($CDCl_3$, 400 MHz): δ (ppm) 7.64 (dd, *J* = 5.0, 2.2 Hz, 2H), 7.29 (dd, *J* = 5.0, 2.2 Hz, 2H), 7.09 (s, 2H), 3.97 (d, *J* = 5.0 Hz, 4H), 1.89 – 1.78 (m, 2H), 1.63 – 1.42 (m, 8H), 1.40 – 1.29 (m, 8H), 0.97 (t, *J* = 7.5 Hz, 6H), 0.91 (t, *J* = 6.0 Hz, 6H). ^{13}C NMR ($CDCl_3$, 100 MHz): δ (ppm) 149.89, 129.24, 126.16, 123.81, 107.47, 71.13, 39.49, 30.72, 29.18, 24.05, 23.11, 14.11, 11.25.

1,4-Dibromo-2,3-bis(2-ethylhexyloxy)naphthalene (EHONBr). To a solution of EHON (1.60 g, 4.16 mmol) in acetic acid (65 mL) was slowly added bromine (3.48 g, 21.8 mmol) at room temperature. The reaction mixture was stirred for 72 h in the dark. Then, water was added and the reaction mixture was extracted with chloroform. The combined organic layers were dried over anhydrous Na_2SO_4 and filtered. The solvent was removed *in vacuo* and the residue was purified by column chromatography on silica gel (*n*-hexane) to afford a yellow oil (1.80 g, 80%). ^{1}H NMR ($CDCl_3$, 400 MHz): δ 8.23 (dd, *J* = 6.4, 3.3 Hz, 2H), 7.53 (dd, *J* = 6.5, 3.3 Hz, 2H), 3.98 (d, *J* = 6.3 Hz, 4H), 1.94 – 1.82 (m, 2H), 1.73 – 1.42 (m, 8H), 1.40 – 1.29 (m, 8H), 0.98 (t, *J* = 7.5 Hz, 6H), 0.92 (t, *J* = 6.9 Hz, 6H). ^{13}C NMR ($CDCl_3$, 100 MHz): δ 150.37, 130.19, 127.24, 126.88, 116.29, 77.42, 40.57, 30.28, 29.18, 23.65, 23.15, 14.14, 11.17.

2,2′-(2,3-Bis(2-ethylhexyloxy)naphthalene-1,4-diyl)dithiophene (EHONDT). A two-necked flask was charged with EHONBr (0.55 g, 1.01 mmol) and $Pd[PPh_3]_4$ (0.059 g, 5% mol). The flask was purged through vacuum/nitrogen filling (3 cycles). Then, anhydrous and oxygen-free toluene (7.0 mL) and 2-(tributylstannyl)thiophene (0.82 g, 2.20 mmol) were added and the reaction mixture was stirred and heated at 110 °C for 48 h under N_2. After cooling to room temperature, water was added and the reaction mixture was extracted with chloroform. The combined organic layers were dried over anhydrous Na_2SO_4 and filtered. The solvent was removed *in vacuo* and the residue was purified by flash chromatography on silica gel (*n*-hexane) to afford a yellow oil (0.54 g, 98 %). ^{1}H NMR ($CDCl_3$, 400 MHz): δ 7.73 (dd, *J* = 6.5, 3.3 Hz, 2H), 7.49 (dd, *J* = 5.1, 1.0 Hz, 2H), 7.32 (dd, *J* = 6.5, 3.3 Hz, 2H), 7.19 (dd, *J* = 5.1, 3.5 Hz, 2H), 7.12 (dd, *J* = 3.4, 1.0 Hz, 2H), 3.79 (d, *J* = 6.2 Hz, 4H), 1.55 – 1.44 (m, 2H), 1.29 – 1.18 (m, 8H), 1.17 – 1.10 (m, 8H), 0.86 (t, *J* = 7.1 Hz, 6H), 0.75 (t, *J* = 7.5 Hz, 6H). ^{13}C NMR ($CDCl_3$, 100 MHz): δ 150.88, 136.13, 131.34, 128.90, 126.69, 126.21, 125.65, 125.61, 125.24, 77.28, 40.33, 30.06, 29.10, 23.30, 23.08, 14.11, 11.04.

5,5′-(2,3-Bis(2-ethylhexyloxy)naphthalene-1,4-diyl)bis(2-bromothiophene) (EHONDTBr). To a solution of EHONDT (0.46 g, 0.83 mmol) in $CHCl_3$/acetic acid (1:1 v/v) (18 mL), NBS (0.33 g, 1.83 mmol) was portion-wise at room temperature. The reaction mixture was stirred for 24 h in the dark. Then, water was added and the reaction mixture was extracted with chloroform. The combined organic layers were dried over anhydrous Na_2SO_4 and filtered. The solvent was removed *in vacuo* and the residue was purified by flash chromatography on silica gel (*n*-hexane) to afford a yellow oil (0.48 g, 81 %). ^{1}H NMR ($CDCl_3$, 400 MHz): δ 7.77 (dd, *J* = 6.3, 3.3 Hz, 2H), 7.36 (dd, *J* = 6.4, 3.2 Hz, 2H), 7.14 (d, *J* = 3.6 Hz, 2H), 6.87 (d, *J* = 3.6 Hz, 2H), 3.79 (d, *J* = 6.2 Hz, 4H), 1.59 – 1.47 (m, 2H), 1.31 – 1.22 (m, 8H), 1.20 – 1.12 (m, 8H), 0.88 (t, *J* = 7.1 Hz, 6H), 0.79 (t, *J* = 7.4 Hz, 6H). ^{13}C NMR ($CDCl_3$, 100 MHz):

δ 151.02, 137.87, 131.11, 129.73, 129.50, 125.80, 125.59, 125.21, 112.83, 77.58, 40.54, 30.28, 29.29, 23.49, 23.24, 14.30, 11.18.

Synthesis of polymers

Polymer A2: EHONBr (179 mg, 0.33 mmol), BTDT (98 mg, 0.33 mmol), $Pd(OAc)_2$ (3.6 mg, 5% mol), P(*o*-OMePh)$_3$ (23 mg, 20% mol), Cs_2CO_3 (321 mg, 0.99 mmol), pivalic acid (34 mg, 0.33 mmol), and anhydrous THF (0.65 mL, 0.4 M). Reaction temperature = 120 °C. Reaction time = 2 hours. Yield = 20 %. ^{1}H NMR in Figure S1.

Polymer A4: EHONBr (129 mg, 0.24 mmol), BTDT (71 mg, 0.24 mmol), $Pd(OAc)_2$ (4.4 mg, 5% mol), P(*o*-OMePh)$_3$ (17 mg, 20% mol), Cs_2CO_3 (233 mg, 0.71 mmol), pivalic acid (24 mg, 0.24 mmol), and anhydrous THF (0.59 mL, 0.4 M). Reaction temperature = 120 °C. Reaction time = 21 hours. Yield = 54 %. ^{1}H NMR in Figure S2.

Polymer B1: EHONDT (186 mg, 0.34 mmol), BTBr (100 mg, 0.34 mmol), $Pd(OAc)_2$ (4.0 mg, 5% mol), P(*o*-OMePh)$_3$ (24 mg, 20% mol), Cs_2CO_3 (332 mg, 1.02 mmol), pivalic acid (35 mg, 0.34 mmol), and anhydrous THF (0.70 mL, 0.5 M). Reaction temperature = 120 °C. Reaction time = 1.5 hours. Yield = 13 %. ^{1}H NMR in Figure S3.

Polymer B2: EHONDT (128 mg, 0.23 mmol), BTBr (69 mg, 0.23 mmol), $Pd_2(dba)_3$•$CHCl_3$ (4.2 mg, 2% mol), P(*o*-OMePh)$_3$ (6.6 mg, 8% mol), Cs_2CO_3 (228 mg, 0.70 mmol), pivalic acid (24 mg, 0.23 mmol), and anhydrous THF (0.58 mL, 0.4 M). Reaction temperature = 120 °C. Reaction time = 2 hours. Yield = 32 %. ^{1}H NMR in Figure S4.

Polymer B3a: EHONDT (111 mg, 0.20 mmol), BTBr (59 mg, 0.20 mmol), $Pd_2(dba)_3$•$CHCl_3$ (3.6 mg, 2% mol), P(*o*-OMePh)$_3$ (5.6 mg, 8% mol), Cs_2CO_3 (197 mg, 0.61 mmol), pivalic acid (21 mg, 0.20 mmol), and anhydrous toluene (0.50 mL, 0.4 M). Reaction temperature = 120 °C. Reaction time = 3 hours. Yield = 72 %. ^{1}H NMR in Figure S5.

Polymer B3b: EHONDT (111 mg, 0.20 mmol), BTBr (59 mg, 0.20 mmol), $Pd_2(dba)_3$•$CHCl_3$ (3.6 mg, 2% mol), P(*o*-OMePh)$_3$ (5.6 mg, 8% mol), Cs_2CO_3 (197 mg, 0.61 mmol), pivalic acid (21 mg, 0.20 mmol), and anhydrous toluene (0.50 mL, 0.4 M). Reaction temperature = 120 °C. Reaction time = 3 hours. Yield = 79 %. ^{1}H NMR in Figure S6.

Polymer B3c: EHONDT (104 mg, 0.19 mmol), BTBr (55 mg, 0.19 mmol), $Pd_2(dba)_3$•$CHCl_3$ (3.4 mg, 2% mol), P(*o*-OMePh)$_3$ (5.2 mg, 8% mol), Cs_2CO_3 (184 mg, 0.57 mmol), pivalic acid (19 mg, 0.19 mmol), and anhydrous toluene (0.47 mL, 0.4 M). Reaction temperature = 120 °C. Reaction time = 5 hours. Yield = 74 %. ^{1}H NMR in Figure S7.

Polymer B4: EHONDT (90 mg, 0.16 mmol), BTBr (48 mg, 0.16 mmol), Pd(Herrmann-Beller) (3.0 mg, 2% mol), P(*o*-OMePh)$_3$ (4.5 mg, 8% mol), Cs_2CO_3 (160 mg, 0.49 mmol), pivalic acid (17 mg, 0.16 mmol), and anhydrous toluene (0.50 mL, 0.4 M). Reaction temperature = 120 °C. Reaction time = 5 hours. Yield = 69 %. ^{1}H NMR in Figure S8.

Polymer B5: EHONDT (150 mg, 0.27 mmol), BTBr (80 mg, 0.27 mmol), $Pd(OAc)_2$ (1.2 mg, 2% mol), P(*o*-OMePh)$_3$ (7.6 mg, 8% mol), Cs_2CO_3 (267 mg, 0.82 mmol), pivalic acid (28 mg, 0.27 mmol), and anhydrous toluene (0.68 mL, 0.4 M). Reaction temperature = 120 °C. Reaction time = 3 hours. Yield = 64 %. ^{1}H NMR in Figure S9.

Polymer B6: EHONDT (114 mg, 0.21 mmol), BTBr (61 mg, 0.21 mmol), $Pd_2(dba)_3$•$CHCl_3$ (3.8 mg, 2% mol), P(*o*-OMePh)$_3$ (3.0 mg, 4% mol), Cs_2CO_3 (203 mg, 0.62 mmol), pivalic acid (21 mg, 0.21 mmol),

and anhydrous toluene (0.52 mL, 0.4 M). Reaction temperature = 120 °C. Reaction time = 5 hours. Yield = 67 %. ^{1}H NMR in Figure S10.

Polymer B7: EHONDT (114 mg, 0.21 mmol), BTBr (61 mg, 0.21 mmol), $Pd_2(dba)_3$•$CHCl_3$ (3.8 mg, 2% mol), P(*o*-OMePh)$_3$ (12 mg, 16% mol), Cs_2CO_3 (203 mg, 0.62 mmol), pivalic acid (21 mg, 0.21 mmol), and anhydrous toluene (0.51 mL, 0.4 M). Reaction temperature = 120 °C. Reaction time = 5 hours. Yield = 38 %. ^{1}H NMR in Figure S11.

Polymer S1 (SPC): A mixture composed of EHONDTBr (177 mg, 0.25 mmol), BTBOR (97 mg, 0.25 mmol), and Pd[PPh_3]$_4$ (12 mg, 4% mol) was purged under a steady stream of N_2 for 30 min at room temperature. Degassed and anhydrous toluene (5.0 mL, 0.05 M) and 2 M K_2CO_3 (0.5 mL) were then added and the mixture was heated at 110 °C for 48 h under the N_2 atmosphere. After cooling to room temperature, the mixture was poured into a 9:1 v/v methanol-acidic water solution and filtered through a Soxhlet thimble. The solid was washed with acetone and *n*-hexane until the wash solution of each extraction was colorless. Then, the solid was extracted with chloroform and the solution was then concentrated to 5-10 mL and precipitated in methanol, filtered through a 0.45 μm nylon filter, and vacuum-dried to afford a red solid. Yield = 18 %. ^{1}H NMR in Figure S12.

^{1}H NMR characterization

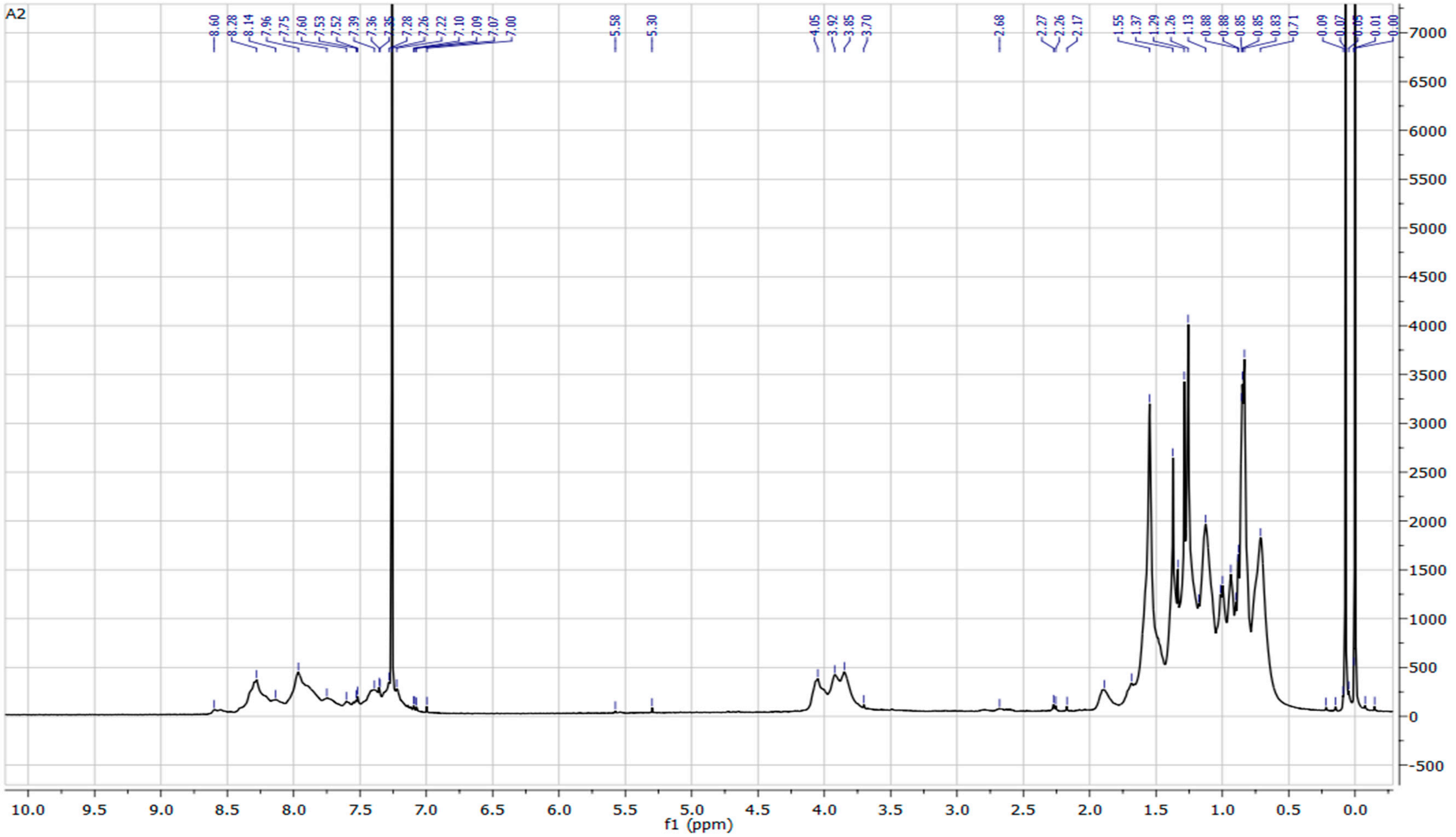

Figure S1. ^{1}H NMR spectra of A2 entry in $CDCl_3$.

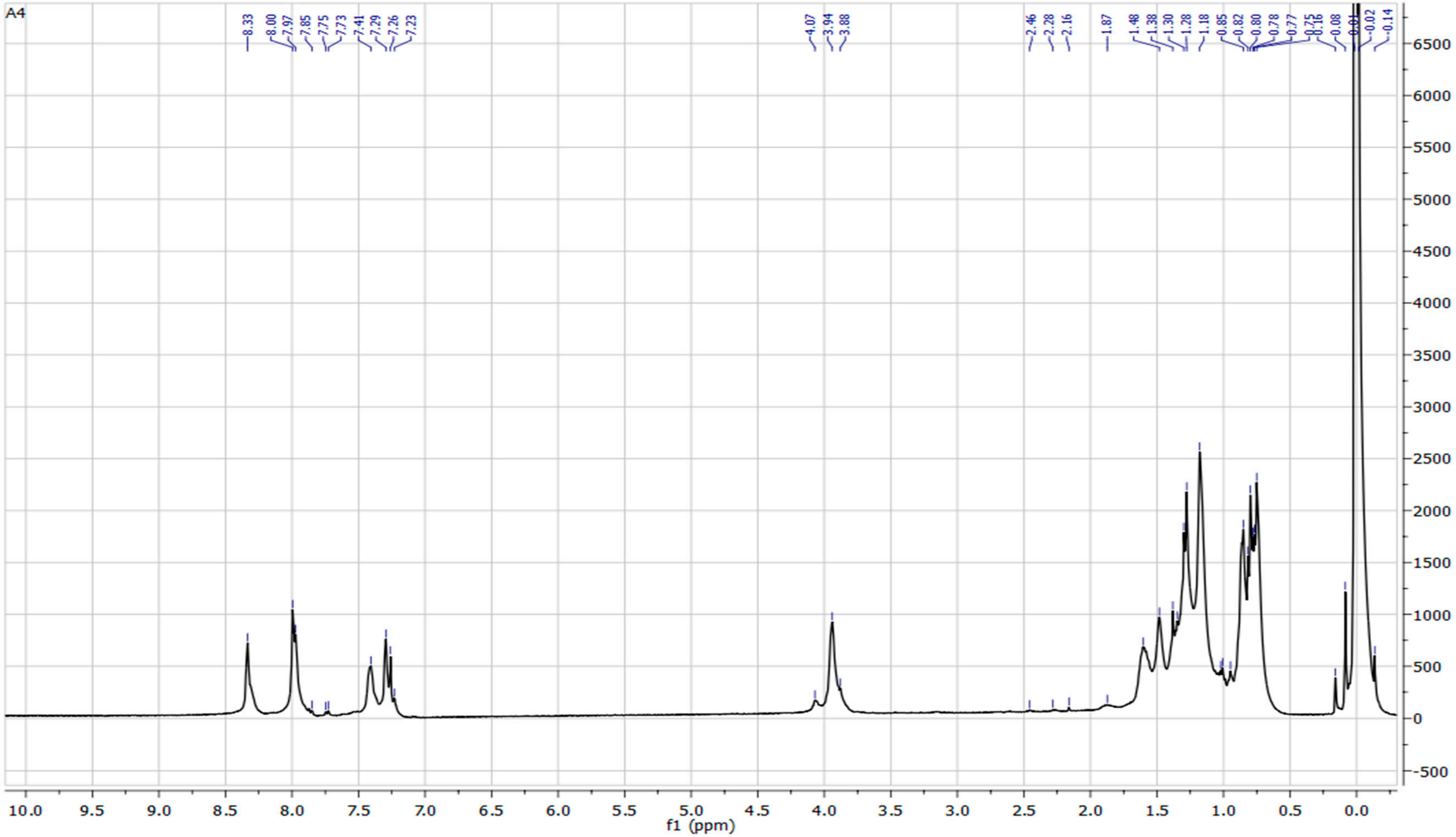

Figure S2. ^{1}H NMR spectra of A4 entry in $CDCl_3$.

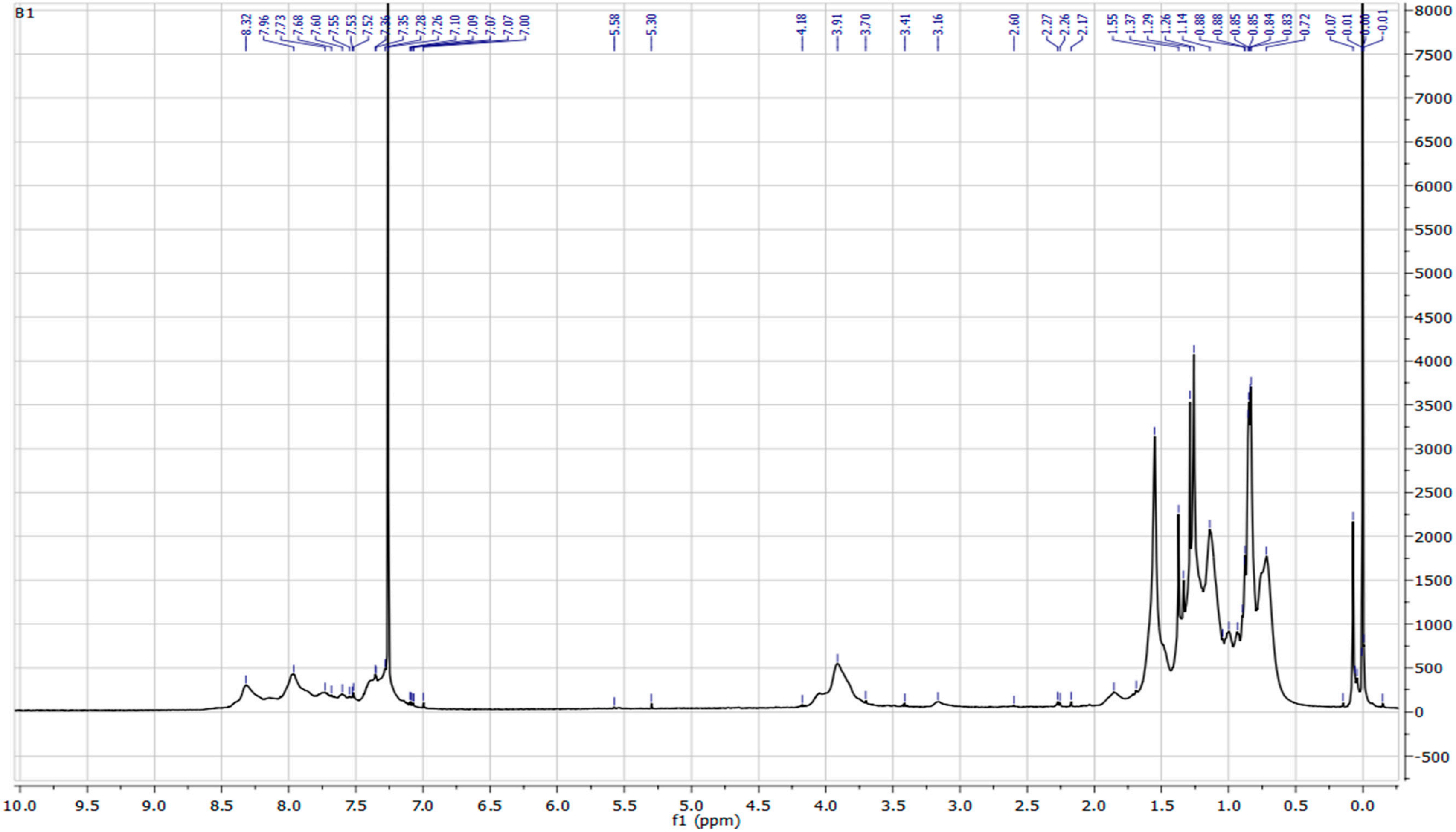

Figure S3. ^{1}H NMR spectra of B1 entry in $CDCl_3$.

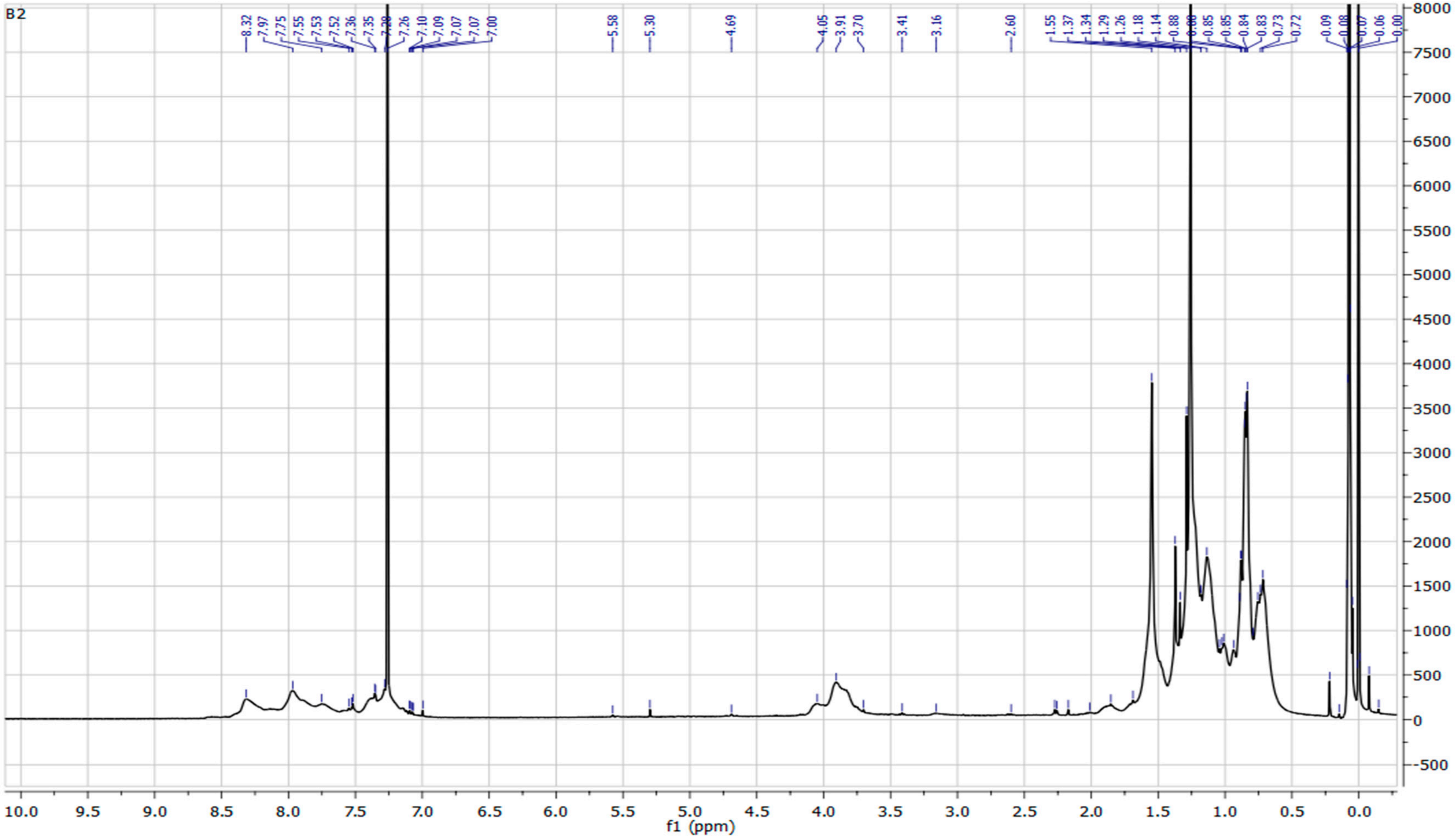

Figure S4. ^{1}H NMR spectra of B2 entry in $CDCl_3$.

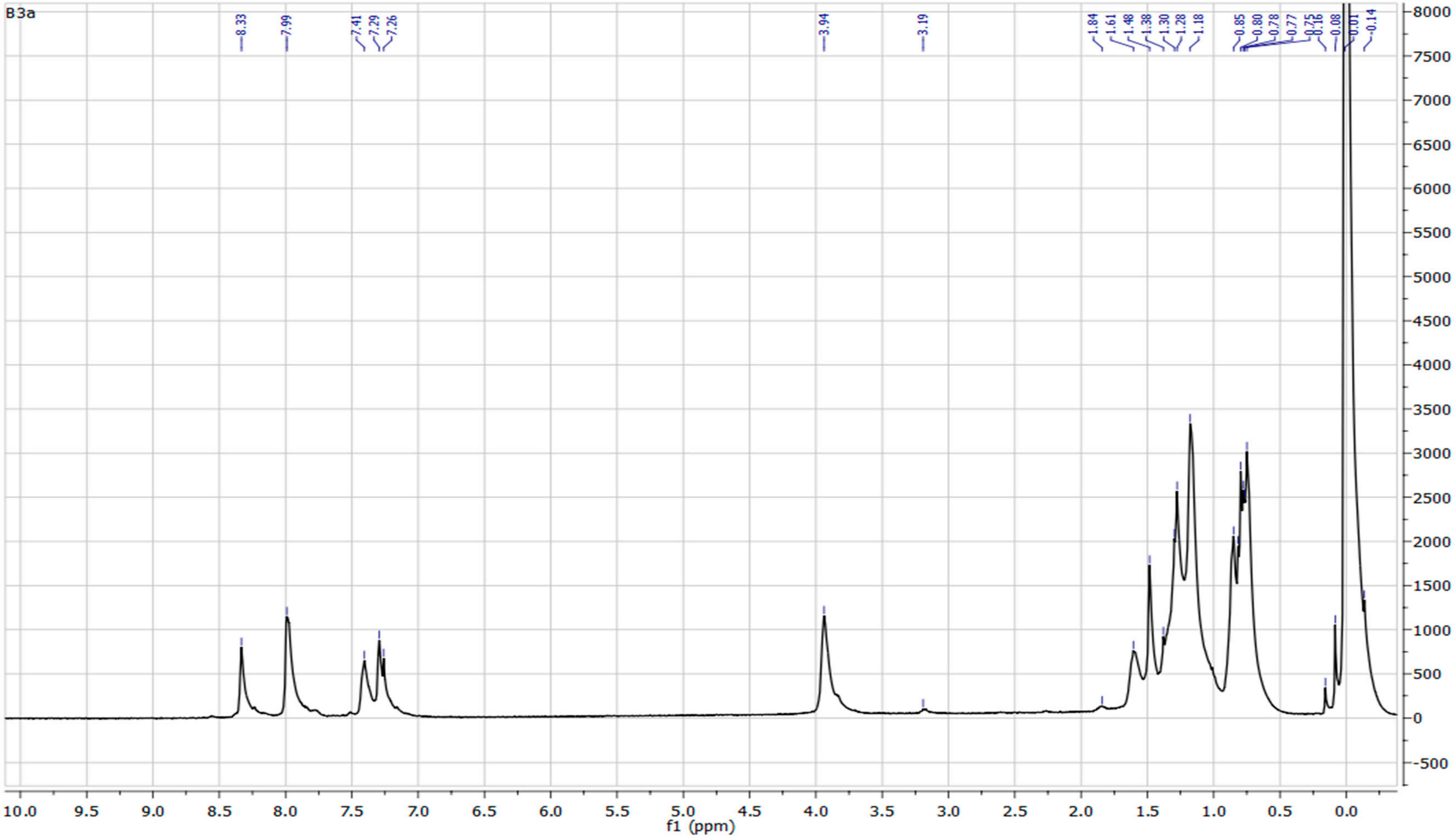

Figure S5. ^{1}H NMR spectra of B3a entry in $CDCl_3$.

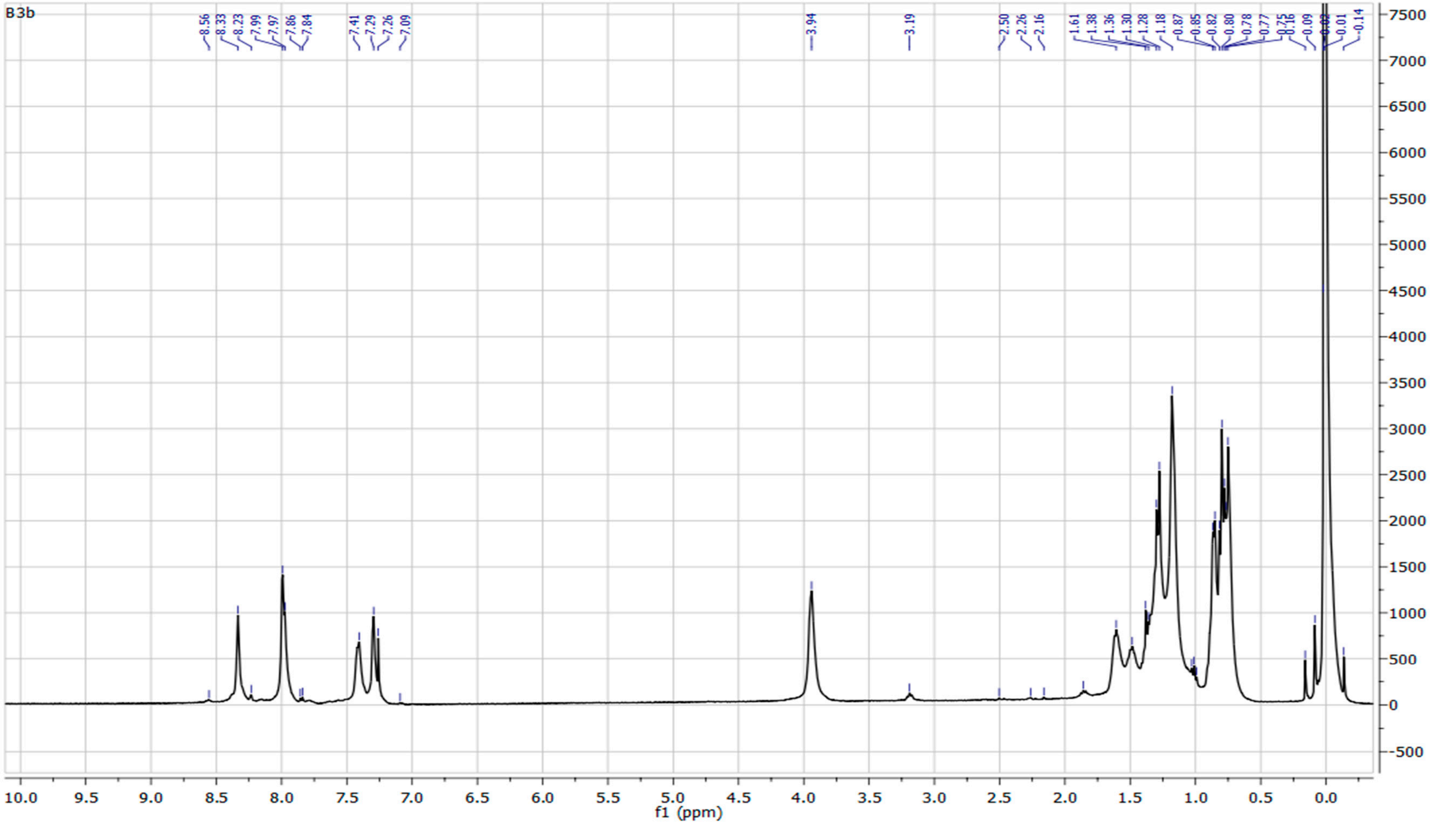

Figure S6. ^{1}H NMR spectra of B3b entry in $CDCl_3$.

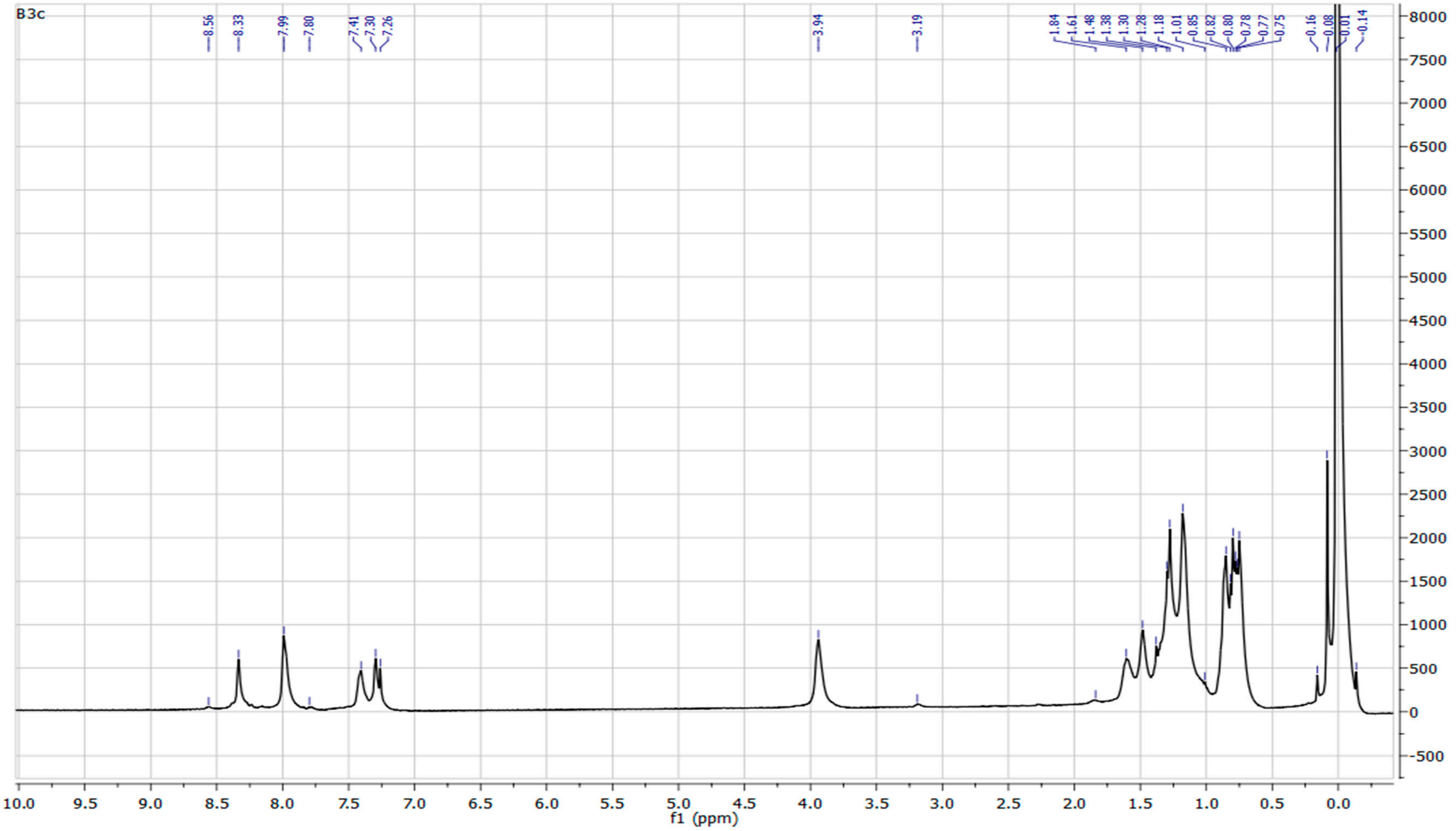

Figure S7. ^{1}H NMR spectra of B3c entry in $CDCl_3$.

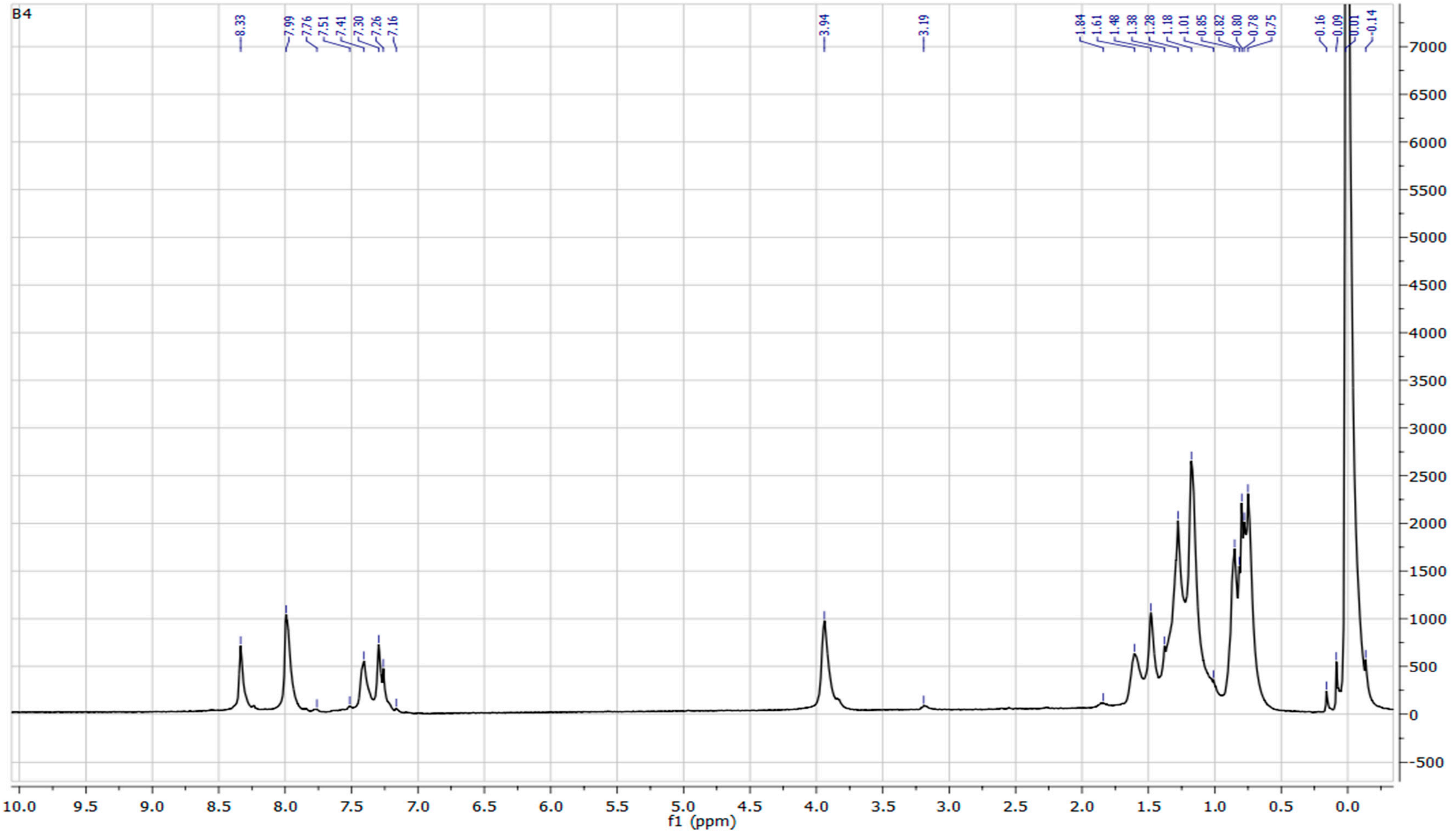

Figure S8. ^{1}H NMR spectra of B4 entry in $CDCl_3$.

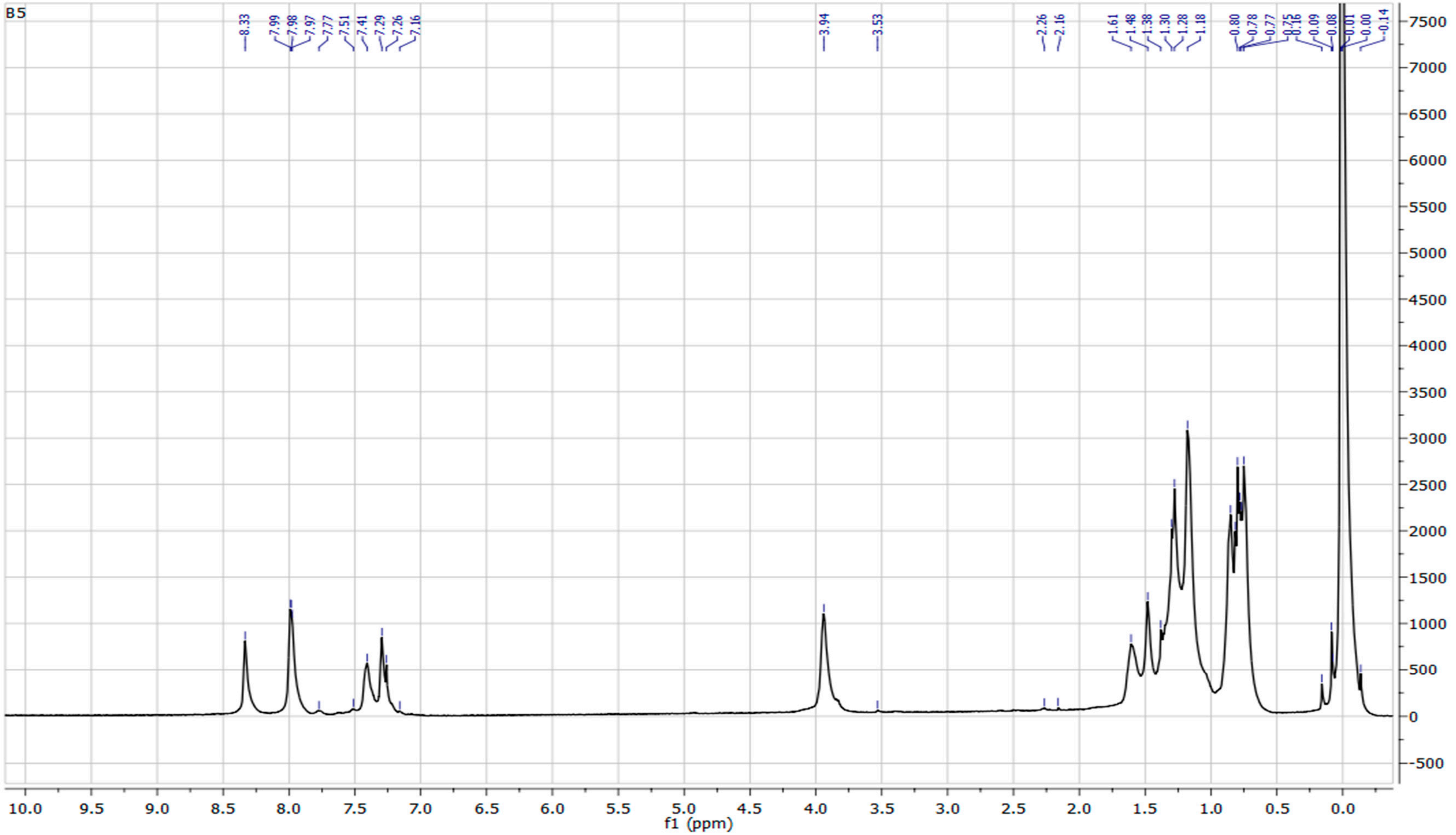

Figure S9. ^1H NMR spectra of B5 entry in $CDCl_3$.

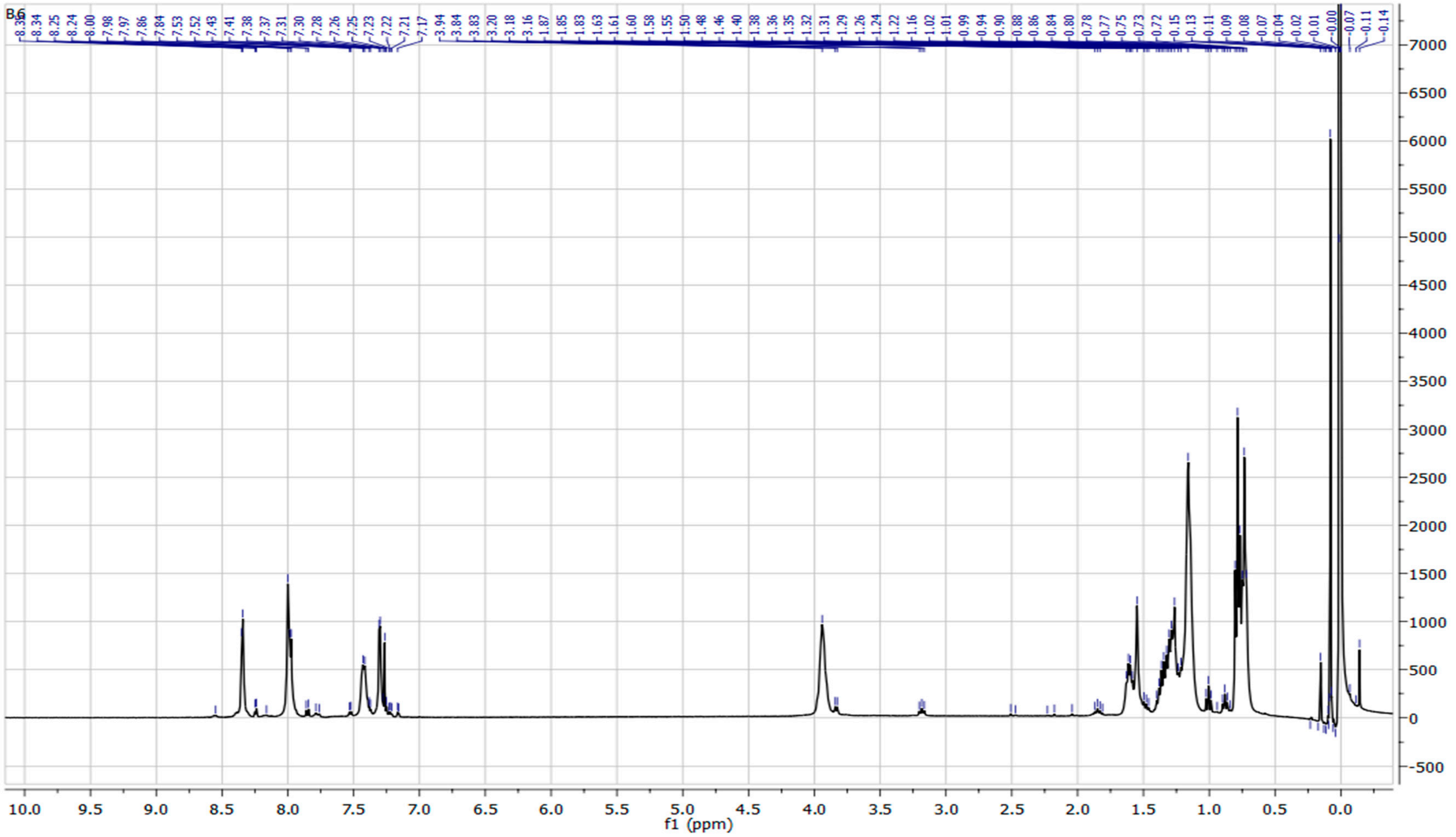

Figure S10. ^{1}H NMR spectra of B6 entry in $CDCl_3$.

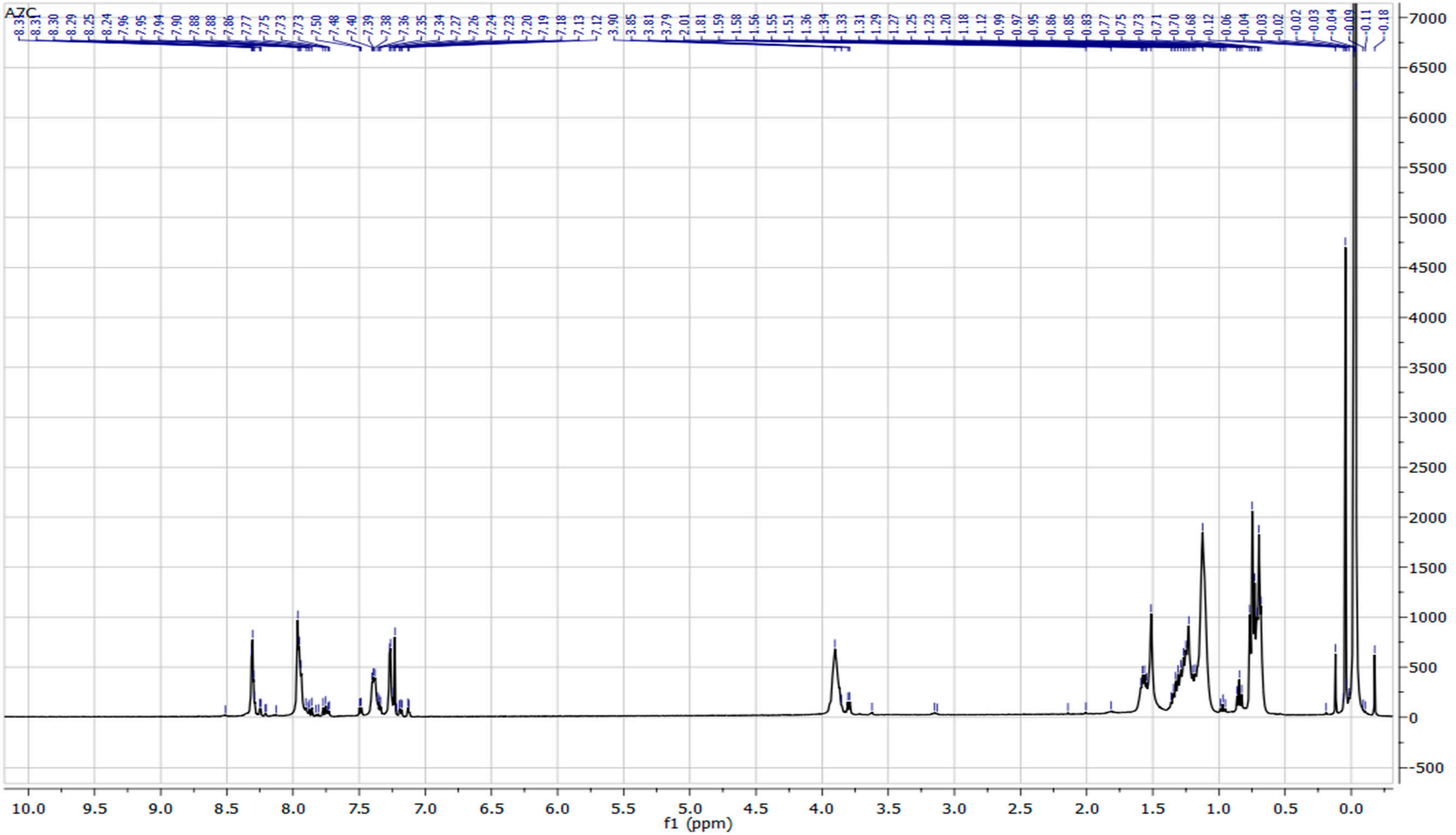

Figure S11. ^{1}H NMR spectra of B7 entry in $CDCl_3$.

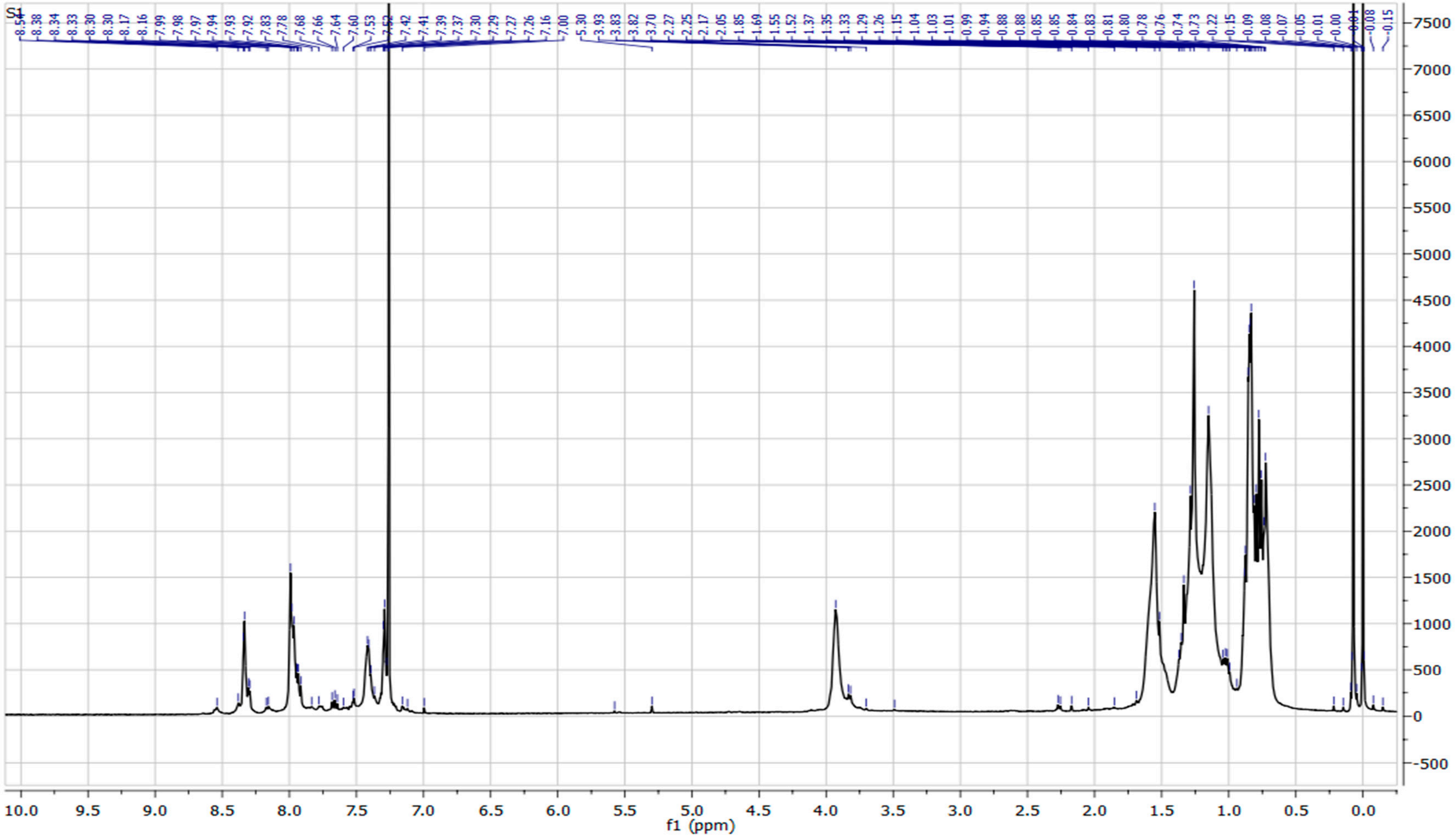

Figure S12. ^1H NMR spectra of S1 entry in $CDCl_3$.

Thermal characterization

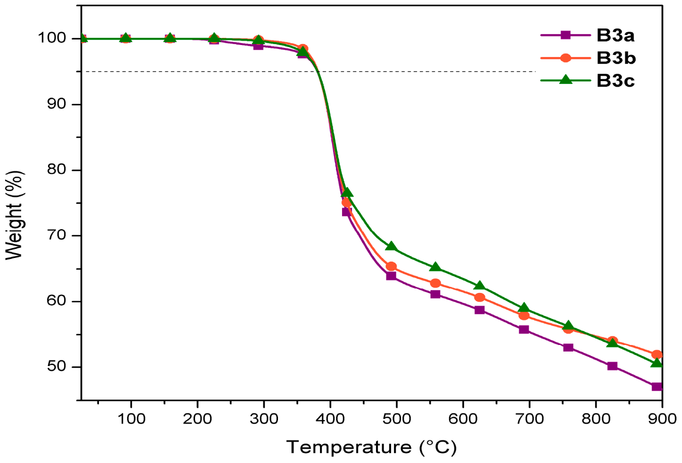

Figure S13. TGA thermograms of B3a-c.

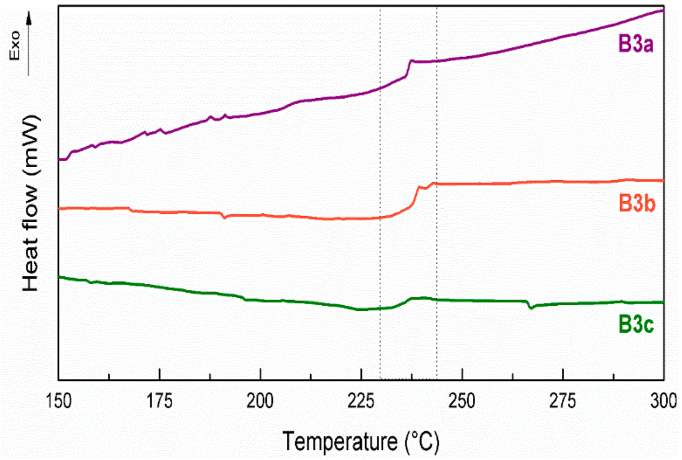

Figure S14. DSC traces for second heating for B3a-c under nitrogen at speed scanning of 10 °C·min^{-1}.

UV-vis characterization

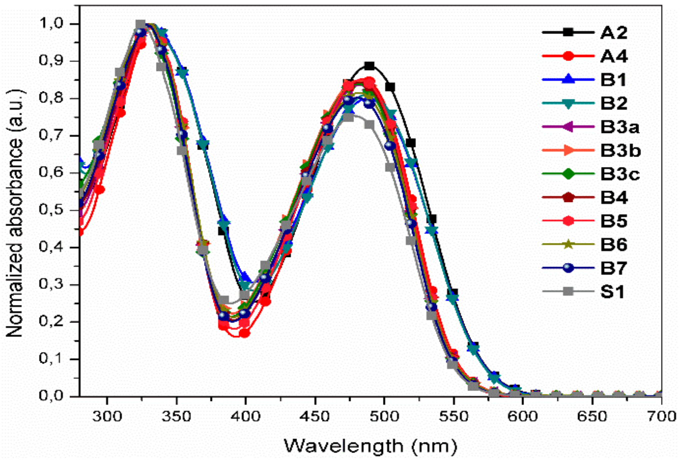

Figure S15. UV-vis of all polymers synthesized in chloroform dilution solution.

Electrochemical characterization

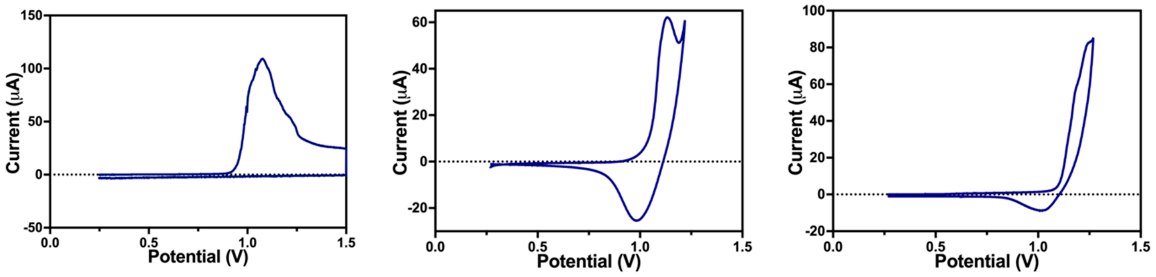

a) **b)** **c)**

Figure S16. Cyclic voltammograms of a) B3a, b) B3b, and c) B3c. Scan rate = 50 m·Vs^{-1}.

Theoretical calculations

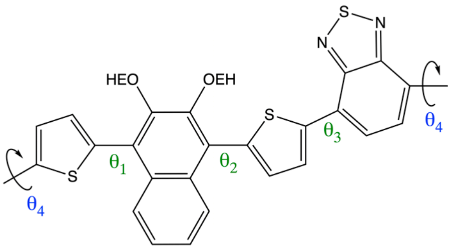

Structure	Θ1	Θ2	Θ3	Θ4
Monomer	73.6	77.4	168.4	-
Dimer	73.6	76.7	168.9	167.7
	69.9	77.1	168.7	
Trimer	73.4	76.8	171.1	170.0
	71.5	76.6	170.4	168.1
	70.5	77.7	168.7	

Figure S17: Dihedral angles of the monomer, dimer, and trimer of PEHONDTBT, determined from theoretical simulations. All calculations were performed at B3LYP/TZVP level.

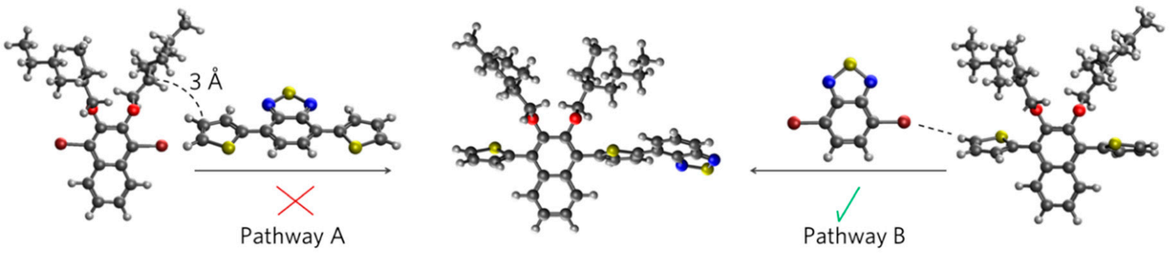

Figure S18: Scheme of the polymerization pathways, highlighting the reduced cavity available for the coupling between the BTDT and the aryl bromide derivative (EHONBr).

Cartesian coordinates for the calculated species

Model catalyst

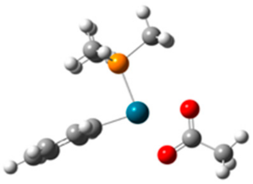

Pd 2.4320193212,3.3047574953,17.7868340983
P 0.8297803616,4.6941737392,18.6582507484
C 2.2594139193,4.3264646911,16.044453265
C 3.1266349274,5.9793167841,14.5020216555
H 3.8068605898,6.7983472074,14.2971540531
C 1.4023825302,3.8674958101,15.0437074744
H 0.7337940378,3.035245607,15.2322158099
C 3.1165520723,5.3918577054,15.7671698686
H 3.7979968967,5.760312643,16.5255006012
C 1.4139314031,4.4562914859,13.7791993615
H 0.7511914897,4.081060469,13.007536899
C 2.2735782256,5.5153792789,13.5058060027
H 2.2823193154,5.9717871009,12.5234159227
O 3.0109175787,1.8845965649,19.4018176976
O 3.9743199495,1.7393318133,17.422608709
C 3.8399455511,1.3288397166,18.6137911433
C 4.6901161229,0.1839131137,19.1031153767
H 4.2941768458,-0.2200572587,20.0329611968
H 4.7440245872,-0.5933561782,18.3408581699
H 5.7060591498,0.5462357602,19.2771568413
C 1.2257137509,6.4924741099,18.7450197342
H 1.3627026009,6.8805437408,17.7365782367
H 0.4240037739,7.0412192204,19.2431092856
H 2.1546135689,6.6312048336,19.298215667
C -0.8387148494,4.6549951413,17.8754656745
H -1.535215071,5.2976665132,18.4175192487
H -0.7601992639,4.9905053867,16.8422253634
H -1.2152026409,3.6320742011,17.879397773
C 0.5066345296,4.226638283,20.4132076425
H -0.254611733,4.8705611358,20.8582832161
H 0.1803823025,3.1877740878,20.4527942772
H 1.4334011563,4.3041267972,20.9809709862

TS for the CMD activation of the BTDT α protons

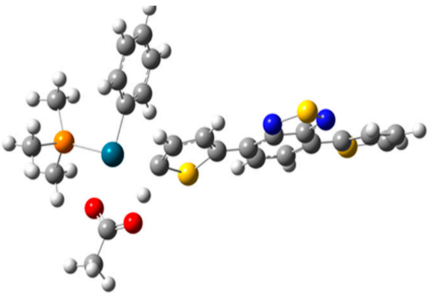

Pd -1.0612626195,-0.0095346433,-0.2992947981

82	C	-0.4722398261,3.9609547307,1.2597585437
83	C	-0.8441345507,2.6270146334,1.0921835054
84	C	-0.4917389853,1.9286007119,-0.0642018286
85	C	0.2395336941,2.5912346934,-1.0500413627
86	C	0.6095762645,3.9260565742,-0.88314697
87	C	0.2534395525,4.6169208178,0.2704323988
88	O	-1.8631332721,-2.0924964448,-0.5375907753
89	C	-1.291116833,-3.0610588334,0.0149783863
90	P	-3.1545499717,0.7012721681,-1.1513039852
91	O	-0.1928460291,-2.9740792834,0.6611456621
92	H	-1.4023582077,2.1355015219,1.8813339829
93	H	1.1830431539,4.4227016984,-1.6579838876
94	H	0.5440672682,5.652408558,0.400327976
95	H	-0.748813185,4.484152078,2.1684960686
96	H	0.5413324979,2.0701792629,-1.9506352721
97	C	-1.9014764066,-4.4399815168,-0.0784519623
98	H	-2.8958920689,-4.3937648814,-0.5159910369
99	H	-1.2597272929,-5.0699916604,-0.6977179864
100	H	-1.9429122106,-4.8934147189,0.91219212
101	H	0.2767203965,-1.84927844,0.5646964254
102	C	-3.572229709,-0.1890470605,-2.714039059
103	H	-4.5943447493,0.0264790824,-3.0323339978
104	H	-2.8796012948,0.1144768855,-3.4993703051
105	H	-3.4532353634,-1.2582123652,-2.5428172999
106	C	-4.555178236,0.25842456,-0.0305415677
107	H	-5.518585846,0.4856527657,-0.4919079648
108	H	-4.4998832884,-0.8065071679,0.1933127538
109	H	-4.4643974763,0.8142788898,0.9028256949
110	C	-3.4739176867,2.4699857445,-1.5595242626
111	H	-4.4860554495,2.6007079416,-1.9472005162
112	H	-3.3400228678,3.0801327521,-0.6671166297
113	H	-2.7519231562,2.8061369301,-2.303101363
114	C	1.5426300531,-0.1227719129,1.5784844885
115	C	2.9067557259,0.2087909964,1.4812713638
116	C	0.9536341941,-0.6279491578,0.4288200046
117	C	3.4405813568,-0.0191360201,0.2262012576
118	S	2.1933480701,-0.6790709885,-0.8208406108
119	H	3.4954346837,0.5975569175,2.2966939338
120	C	4.7935217093,0.2090375681,-0.2549458896
121	C	5.118323217,0.1429198923,-1.5966546796
122	C	5.8974995004,0.5166319659,0.6180702107
123	C	6.4172343971,0.3460629101,-2.1037103651
124	C	7.2405210445,0.7402420833,0.0906843469

C 7.5122715579,0.6528226848,-1.3197785627
N 5.8320299173,0.6294050997,1.9420422946
N 8.1352505078,1.0172399456,1.0360976086
S 7.3441194274,0.9935816745,2.480217029
H 0.984723826,-0.0050748387,2.4991517407
H 4.3362569993,-0.0754333621,-2.312663027
H 6.548030396,0.2498317615,-3.1744835772
C 8.8374806788,0.8743941937,-1.8792793193
C 10.0570047776,0.9579960228,-1.2489882656
S 9.0541124618,1.0680004017,-3.6224590212
C 11.1477628682,1.1617747701,-2.1337638947
H 10.1556911362,0.8775352465,-0.1786374341
C 10.7704573542,1.2403493575,-3.440483744
H 12.1745562323,1.2473197232,-1.8063310624
H 11.3879346623,1.3938870806,-4.3108845125

TS for the CMD activation of the BTDT β protons

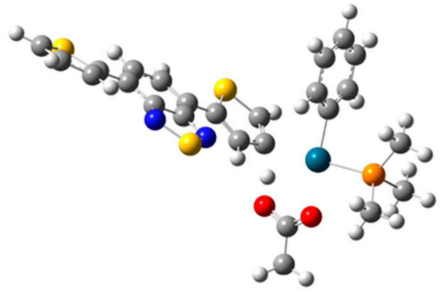

Pd 0.8459727039,0.2560583289,-0.2805924912
C 2.1172728817,3.8376211117,-2.3113773274
C 1.7819738035,2.5197625511,-1.999058115
C 1.3118480539,2.1855709163,-0.7274875717
C 1.1831062671,3.1959281044,0.2257901853
C 1.5216872737,4.5128721312,-0.0857670411
C 1.9924763522,4.8389220596,-1.3537053969
O 0.4960805622,-1.9158315409,0.1567347318
C -0.5877469477,-2.4401362513,-0.1894145989
P 3.1286261115,-0.1908027909,0.1642253839
O -1.564102478,-1.7927909251,-0.6994362455
H 1.8821645521,1.7582003653,-2.7648096624
H 1.4093332917,5.2856348287,0.6666047255
H 2.2515168043,5.8627374576,-1.5951492343
H 2.474046786,4.0784550107,-3.3067998051
H 0.8016878479,2.9703515942,1.2144941905
C -0.7972446825,-3.9232948431,0.0030414476
H -1.16508777,-4.3671269202,-0.9224560799

H 0.1257765964,-4.4067875552,0.3138056493
H -1.5644363993,-4.0773832474,0.7644780443
H -1.3344760466,-0.5956774352,-0.6803659092
C 3.3289668657,-1.1508898982,1.7286453291
H 4.360988089,-1.4823520841,1.8608953385
H 3.0407521455,-0.5264248178,2.5746101284
H 2.6621018377,-2.0113321028,1.6924096616
C 3.8947667488,-1.2942228742,-1.1050161874
H 4.9118849556,-1.5752883348,-0.8236897749
H 3.2838598626,-2.1910032571,-1.205142802
H 3.9174601172,-0.7812668842,-2.066655935
C 4.3524411469,1.176752908,0.3363134322
H 5.3466796304,0.7809079004,0.5522279193
H 4.3798021573,1.759972352,-0.5831858223
H 4.0413124822,1.8407997348,1.1422745776
C -1.3111918439,0.8164332882,-0.5642201485
C -1.9729402171,1.3504233743,0.5930939752
C -1.7343321108,1.4785542546,-1.6921764393
C -2.8576904647,2.3726131565,0.3556461326
S -2.9009636743,2.7200989863,-1.3788955872
H -1.8088774194,0.9760412515,1.59193335
H -1.4243725032,1.3167715072,-2.7137111004
C -3.705974659,3.1112531483,1.2784316806
C -4.776832876,3.8750071464,0.8575450705
C -3.4904377509,3.107804633,2.7024962264
C -5.6108931828,4.6082249998,1.7274337578
C -4.3638723396,3.854372899,3.603601558
C -5.4649246453,4.6318367635,3.0998210772
N -2.5177852094,2.4579976078,3.3365604498
N -4.0210219234,3.7343567198,4.8838674027
S -2.6953342143,2.7584490517,4.9457675225
H -5.0062768271,3.9183704514,-0.1996471693
H -6.4095925223,5.1824831351,1.2742965393
C -6.3592096207,5.3824224226,3.9702317573
C -6.2249330945,5.7109337369,5.2985799748
S -7.8768274226,6.0373200321,3.3457194173
C -7.3024859536,6.4795338925,5.8118455703
H -5.3737671942,5.4009058319,5.8827789025
C -8.2661075249,6.7379267089,4.8844120971
H -7.3556208814,6.8250017404,6.8349526199
H -9.1844255276,7.290026668,5.0039406184

TS for the CMD activation of the BTDT γ protons

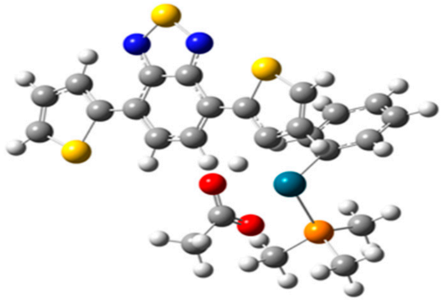

Pd 0.9630742397,0.5366595885,-0.3423721241
C 2.4458630606,4.0737609566,-2.3151243139
C 2.0085959566,2.7785446653,-2.0364455414
C 1.5553873651,2.4393240474,-0.7612508801
C 1.5492376087,3.4207770866,0.2304850655
C 1.9886433649,4.7151713733,-0.0474994354
C 2.4420007745,5.0460579892,-1.3203440596
O 0.4624087269,-1.6044517211,0.1307080974
C -0.6907511419,-2.0427824057,-0.082799591
P 3.2009572103,-0.0615749263,0.1617566782
O -1.6515876271,-1.3361102788,-0.5440143392
H 2.0123833583,2.042300383,-2.8319753135
H 1.9696429832,5.4660997175,0.7346355843
H 2.7801242709,6.0522351936,-1.5368577779
H 2.7863688729,4.319901215,-3.3148143247
H 1.1875393068,3.1919891342,1.2260118984
C -1.0123656829,-3.4885116871,0.2112053922
H -1.7795238739,-3.5347018341,0.9861515145
H -1.4258282907,-3.9598874609,-0.6813394174
H -0.1239784129,-4.0220950688,0.5400261805
H -1.3160773908,-0.1788672009,-0.6532092574
C 3.8287289395,-1.4111263928,-0.9334704004
H 4.8102596069,-1.7618232261,-0.6075757479
H 3.1187961536,-2.2373563547,-0.9094683747
H 3.901317809,-1.0444459536,-1.957563543
C 4.5597612991,1.183020806,0.1319810607
H 5.5118607306,0.7225469512,0.4025672435
H 4.6322819246,1.6192747067,-0.8635613248
H 4.3299660931,1.9874498145,0.8298935459
C 3.3306589162,-0.8047204408,1.8479723405
H 4.3318451865,-1.2004674823,2.0311655748
H 3.1017708895,-0.0464136806,2.5970515726
H 2.5964336553,-1.6054028362,1.9302824838
C -1.1393732111,1.2323897667,-0.7064971699
C -1.7066596824,1.8873305595,0.4346676276
C -1.5984819684,1.8120286661,-1.8920203438

C -2.5424543226,2.9193573503,0.1421237393
S -2.7048202475,3.150304064,-1.5668189153
H -1.5008190897,1.5777259466,1.451589662
H -3.0853958532,3.5588390804,0.8215794245
C -1.2960804954,1.3885024196,-3.253359614
C -0.4601415227,0.3175447026,-3.5140493594
C -1.8295393725,2.0206023687,-4.4311656375
C -0.1505230165,-0.1443504208,-4.8077362445
H -0.0031846714,-0.1972066911,-2.6803399141
C -1.5112741328,1.5430927007,-5.7731268036
N -2.6467475655,3.0683321199,-4.4315695068
C -0.640829796,0.4162122444,-5.9720559318
H 0.5229335289,-0.9900782265,-4.8771916509
N -2.1009558016,2.2510638921,-6.7338189743
S -2.9891709203,3.4287869811,-5.999866148
C -0.3015454915,-0.094317132,-7.2916206229
C -0.5467746852,0.4428457076,-8.5338155339
S 0.5665998653,-1.6244854907,-7.4669924593
C -0.0475932269,-0.3400548913,-9.6074233206
H -1.0760488372,1.3733400167,-8.6604412315
C 0.5768309702,-1.4786135135,-9.1960621737
H -0.1523689667,-0.0633326054,-10.6473041193
H 1.0387786292,-2.2468582937,-9.7950862442

TS for the CMD activation of the BTDT bencene (C5) protons

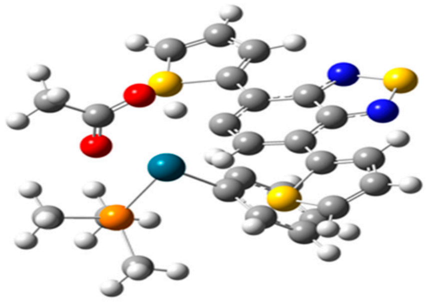

Pd -1.2694859603,-0.1557656773,-0.0200202227
C -0.2073771491,3.6190391815,1.8008226901
C -0.743349411,2.3546318785,1.5544097761
C -0.5189032211,1.7124628896,0.3362395052
C 0.2480186953,2.3620199312,-0.6326790493
C 0.7832731485,3.6264538278,-0.386249502
C 0.5546827342,4.2611420925,0.8303900496
O -2.1826263777,-2.1417264698,-0.4839974133
C -1.5896674624,-3.200878555,-0.1816948233
P -3.2078011972,0.9266660859,-0.8246224434

O -0.4393758543,-3.2443285962,0.3760767615
H -1.3196418136,1.8674485024,2.3320360987
H 1.3852081386,4.1094871358,-1.1477276911
H 0.972886848,5.241763797,1.0229538942
H -0.3835757279,4.0966121672,2.7581826644
H 0.4519103842,1.8843108141,-1.583728586
C -2.2388683308,-4.5336403875,-0.4663583616
H -3.1665287101,-4.3997371954,-1.0172893409
H -1.5513900837,-5.1641348096,-1.0311847013
H -2.4414861888,-5.0398892831,0.4793470024
C 0.7742614403,-0.9419781884,0.658875968
C 3.4929008192,-0.1827227481,0.8157065624
H -0.017487464,-2.1255510665,0.5424522045
C 2.6657941177,-0.2909932908,2.0131972491
C 1.2732643001,-0.6697627027,1.9341611855
C -2.9780196334,2.0965773209,-2.2328624677
H -3.9343936695,2.5241905955,-2.5402747454
H -2.2993828278,2.8937678183,-1.9331345939
H -2.5345465078,1.5655555747,-3.0754417246
C -4.4791398373,-0.2509086342,-1.4596702789
H -5.3607390627,0.2860913479,-1.8152530942
H -4.0516184776,-0.8393412569,-2.2702057326
H -4.7593390441,-0.9404593474,-0.6649022914
C -4.1431342504,1.9305728327,0.4091984383
H -5.0394834979,2.3609706267,-0.041719101
H -4.4304729208,1.2956556925,1.2474726168
H -3.5037269933,2.7283984849,0.784296782
C 1.6379395019,-0.8876303301,-0.4765871094
C 2.9617840069,-0.5095192933,-0.4786104071
H 1.2056716261,-1.195554277,-1.4232263741
N 3.3264502255,0.0295825072,3.1242891254
N 4.7382064218,0.2100124081,1.0649337395
S 4.8629462957,0.4326564078,2.6939637392
C 3.7629760093,-0.4514814266,-1.6947449505
C 5.1223305088,-0.3238933943,-1.8512837701
S 2.9903846195,-0.5588850073,-3.2812591374
C 5.5486215033,-0.323393078,-3.2062125622
H 5.788787848,-0.2284520665,-1.0094250651
C 4.5212757649,-0.4429233316,-4.091983416
H 6.5838580023,-0.2367705819,-3.5061483714
H 4.5606810035,-0.4690217232,-5.1691026311
C 0.4616796215,-0.7808337677,3.139205059
C 0.8183293552,-0.6328602877,4.4627130529

S -1.2550456593,-1.2008004432,3.0416834616
C -0.2416497366,-0.8487112442,5.3787636209
H 1.8219157297,-0.3764986415,4.7577028143
C -1.4188363201,-1.1623076332,4.7680394177
H -0.1274187411,-0.7738066484,6.4514751671
H -2.3738725382,-1.3769675363,5.2207713126

TS for the CMD activation of the EHONDT α protons

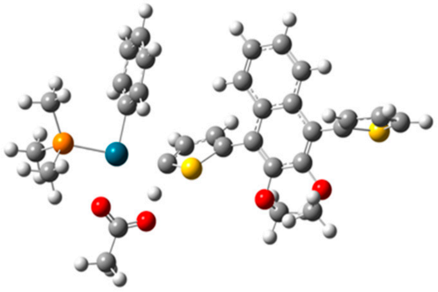

Pd -0.8940785966,0.2099984658,-0.6458706843
C 0.112895085,4.1813732494,0.7095703042
C -0.1344485477,2.8091815142,0.6522656267
C -0.4933432899,2.2010031059,-0.5510783568
C -0.5947213883,2.993246495,-1.6966731729
C -0.3448571938,4.3643424624,-1.6402769668
C 0.0062850804,4.9649101781,-0.4351030827
O -1.5037708501,-1.9454356951,-0.8072831555
C -0.7070635556,-2.8029162943,-1.2535436952
P -3.2043778018,0.6454821711,-0.3428344156
O 0.5080675471,-2.5617682805,-1.566839714
H -0.0297117071,2.2182690965,1.5541058122
H -0.4231621194,4.9609047598,-2.5425383729
H 0.198611082,6.0301669999,-0.3899671193
H 0.3945222253,4.6340804469,1.6537962028
H -0.8595856083,2.5478306209,-2.6491857985
C -1.1698572177,-4.2297818566,-1.4333945686
H -0.6624823971,-4.860704591,-0.7006409066
H -2.2456021731,-4.3074922524,-1.2948278172
H -0.8868338225,-4.5889587175,-2.4230631673
H 0.8130409215,-1.4250250527,-1.2607773758
C -3.8181059724,2.3341042709,0.0691887431
H -4.9039883814,2.3347908229,0.1816494706
H -3.3546970308,2.6717414574,0.9957215667
H -3.5297597546,3.0291074709,-0.7185574953
C -4.2075613371,0.172417381,-1.8207878475
H -5.2771603606,0.2630461895,-1.6198018689

H -3.9444478289,0.8159789448,-2.6604659995
H -3.9716039216,-0.8576170661,-2.086404636
C -3.9110600934,-0.4121643444,0.9958574147
H -4.998075619,-0.318954311,1.0431933455
H -3.6351646331,-1.4472958233,0.7981702326
H -3.482810255,-0.1172428542,1.9540991117
C 4.5910533132,4.6462164967,1.1439339296
C 4.2462566584,3.3526626508,0.8428280888
C 5.0723759447,2.2643649132,1.2229626183
C 6.2636195006,2.5374229705,1.9679731225
C 6.5960658317,3.8880493003,2.2440011315
C 5.7846689222,4.9177199115,1.8392057477
H 3.9421086764,5.4594929928,0.8429146205
H 3.3277917359,3.1504338395,0.3102196766
C 4.7399573145,0.9113088514,0.8850735891
C 7.0851962466,1.4530977303,2.4163193042
H 7.5080892593,4.0975454014,2.7852812854
H 6.0609476086,5.9416917019,2.0601249017
C 6.7238778526,0.160845073,2.0951217895
C 5.5622828654,-0.1088029051,1.3193703026
O 7.5223385688,-0.8904161219,2.4711437586
O 5.2486545039,-1.4173556494,1.048616508
C 7.0303760072,-1.6710337431,3.5703463946
H 6.0557456903,-2.1034085416,3.3376979319
H 6.9643740677,-1.057566086,4.4730544092
C 5.9815787802,-2.0096314604,-0.0338949888
H 7.0538003553,-2.005285467,0.1726115497
H 5.7761313252,-1.4809225943,-0.9682950341
C 8.2936438847,1.6990735343,3.2311532411
C 8.3705398688,2.1134972412,4.5319915253
S 9.90301688,1.5075239156,2.5609240662
C 9.7035282633,2.2763886153,5.0053010557
H 7.4917553535,2.2987716125,5.1348108632
C 10.638877994,1.9915947259,4.0566367877
H 9.9503420126,2.5914709473,6.0097710864
H 11.7131533579,2.0280387322,4.1431259183
C 3.5544905802,0.6053234835,0.0584354116
C 3.3517441455,0.924590398,-1.2643391378
S 2.1621127332,-0.2364326715,0.6824799375
C 2.1008501732,0.4950203054,-1.7582941444
H 4.0918984149,1.4454053828,-1.8577595469
C 1.2885521841,-0.1619280174,-0.8469757752
H 1.7971131886,0.6533204865,-2.7857765629

H 5.6250837627,-3.0345300289,-0.1152886379
H 7.7584382895,-2.4646765114,3.7268557413

TS for the CMD activation of the EHONDT β protons

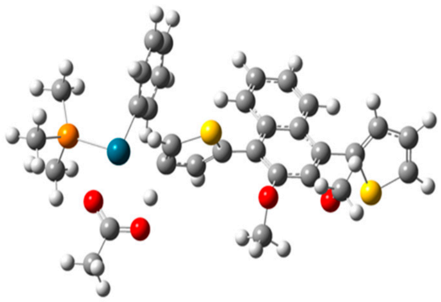

Pd 0.8759152931,0.129483149,-0.2821190728
C 1.6694928379,4.3386965988,-0.805934064
C 1.3269755057,3.0045248454,-1.0252704053
C 1.2902136253,2.0944365432,0.0326818855
C 1.5917813983,2.5525744024,1.317100577
C 1.9356859998,3.8868310798,1.5372104493
C 1.9818717805,4.784117589,0.4749616183
O 0.5995724685,-2.0804522672,-0.5539918849
C -0.5026405135,-2.596197429,-0.2531921574
P 3.1993919371,-0.3426044097,-0.3823792892
O -1.5319550002,-1.9269151671,0.0959224869
H 1.0802705973,2.684049826,-2.0303777724
H 2.1639399082,4.2217007427,2.5430130228
H 2.2518900723,5.8197567207,0.6433462479
H 1.6883469364,5.0305793624,-1.6406449675
H 1.5576405935,1.8747775208,2.1628203199
C -0.6651023662,-4.0970844848,-0.3067382844
H -1.3758765688,-4.3506166249,-1.0956378611
H 0.2875321042,-4.5826794957,-0.5041726709
H -1.0841441462,-4.4550985047,0.6342348731
H -1.3172744815,-0.7243200136,-0.0453752085
C 3.7812496247,-1.326548043,1.0697585254
H 4.8249989432,-1.6260742025,0.9534685628
H 3.6777628827,-0.7314084351,1.9772176051
H 3.1561282693,-2.2140096274,1.1646237848
C 3.6041240228,-1.4356281304,-1.8149611998
H 4.6426130498,-1.7710949219,-1.776802247
H 2.9337193908,-2.2937945589,-1.7933124217
H 3.4370065216,-0.8901510252,-2.7440872823
C 4.4464197525,1.0096155179,-0.507137373
H 5.4574460838,0.6002760471,-0.5525368482

H 4.2518866204,1.6001694355,-1.4019748269
H 4.3556154901,1.6706149296,0.3538570393
C -1.50266774,5.8606333827,2.3008544949
C -1.9707801664,4.7453074064,1.6532428064
C -3.320768966,4.3332709834,1.7854254104
C -4.203060168,5.1220891368,2.5912233503
C -3.6779290668,6.2571058537,3.2593602129
C -2.3614522538,6.6176751112,3.1201399596
H -0.4670759754,6.1531779727,2.1819699252
H -1.304660842,4.1618594464,1.0334358586
C -3.814485727,3.1566958878,1.1351304869
C -5.5821911081,4.7522046261,2.7051227858
H -4.3366048709,6.8411567215,3.8867930428
H -1.9826803933,7.4888317517,3.6411751581
C -6.0292131036,3.6207204899,2.0541985501
C -5.1443231015,2.8178353,1.2825599894
O -7.3344013931,3.2172385249,2.1909819604
O -5.6568264973,1.7208718296,0.6348004973
C -8.177177349,3.4760931768,1.0588490335
H -7.8002582228,2.9717785294,0.1673964716
H -8.2531519515,4.5519584944,0.8789469546
C -5.8189222106,0.5512996954,1.451158555
H -6.493481976,0.7500956567,2.2865263838
H -4.8500439944,0.2069184267,1.8212024438
C -6.529191129,5.573612907,3.4883883625
C -7.0233668023,6.813886772,3.1926817343
S -7.1539987211,5.0260458139,5.0330431075
C -7.9023700392,7.3338931813,4.1848215524
H -6.7613453762,7.3416244472,2.2856003251
C -8.0679541527,6.4872948141,5.2392650549
H -8.3886425017,8.2968930197,4.1117045298
H -8.6745896105,6.6252886669,6.1200797669
C -2.9264132242,2.285082324,0.335581296
C -1.9567497308,1.4269393988,0.7628030605
S -3.0596086994,2.2164491944,-1.4174276881
C -1.300921926,0.6649557542,-0.2694017736
H -1.7252209125,1.3149843944,1.8145333341
C -1.8112482783,1.0164415159,-1.4988875733
H -6.2479540559,-0.2117658362,0.804654412
H -9.1590959491,3.0840699378,1.3170890545
H -1.5494274371,0.6115987412,-2.4657570178

TS for the CMD activation of the EHONDT γ protons

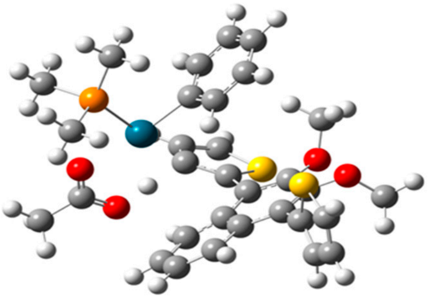

Pd	0.8980256544,0.6170715455,-0.4522185198
C	2.9879474086,3.4922190209,-2.8873741606
C	2.2828884859,2.3707223051,-2.4500324673
C	1.7384331353,2.3250767186,-1.1643603616
C	1.8922879487,3.4380291287,-0.3352065281
C	2.5948006915,4.5614185339,-0.7736639814
C	3.1546627632,4.5890457012,-2.047614155
O	0.2522653145,-1.346322044,0.4375653962
C	-0.965165135,-1.6207023939,0.5225505269
P	3.0697660572,-0.2356466643,-0.0068750256
O	-1.896142356,-0.8503608334,0.1102457124
H	2.1658026454,1.5296439238,-3.1237335643
H	2.7036101665,5.4152114598,-0.1138728574
H	3.7058929838,5.4585884089,-2.3849782615
H	3.4030493541,3.5058155513,-3.8889823589
H	1.4631860917,3.4415998081,0.6597021088
C	-1.4024296704,-2.9269821575,1.1420802233
H	-2.0829229765,-2.7269735194,1.9709143722
H	-1.9548183753,-3.510485117,0.4036434189
H	-0.5438827254,-3.4946397094,1.4924281159
H	-1.4252552244,0.1899039233,-0.3610556414
C	4.5940957852,0.7535382748,-0.3171555545
H	5.4818976373,0.1940123019,-0.0162294874
H	4.6603389189,1.0045606601,-1.3746842526
H	4.5419314037,1.6864871928,0.2432981391
C	3.2383155719,-0.7240742866,1.7665011638
H	4.1832242346,-1.2415979121,1.9450814847
H	3.192915361,0.1667596876,2.3933879532
H	2.4048028418,-1.3752185453,2.0258388238
C	3.3880462892,-1.8155375529,-0.9089070893
H	4.3313531313,-2.2675878531,-0.5950098789
H	2.5658354398,-2.5008320164,-0.7063908117
H	3.4206222805,-1.6218731026,-1.98116239
C	-1.5435606751,-1.933628633,-3.4728649418
C	-1.6959888944,-0.6430660508,-3.030118543
C	-1.2034658157,0.4606727572,-3.7660223993

C	-0.6268130797,0.1993319505,-5.0510186307
C	-0.4400509019,-1.1469126228,-5.4564813891
C	-0.8756868463,-2.1925242327,-4.68283948
H	-1.9493569848,-2.7519343403,-2.8903488743
H	-2.2330689658,-0.4631987031,-2.1151453993
C	-1.3263718142,1.8135769907,-3.2832163476
C	-0.2934051416,1.2884084862,-5.9165513944
H	0.0362413045,-1.3384900958,-6.4077822795
H	-0.7315759694,-3.2124782757,-5.0186720338
C	-0.5597962591,2.5711925092,-5.4903139045
C	-1.0663940049,2.8359086535,-4.1888247882
O	-0.2716874155,3.6386004035,-6.3066499169
O	-1.3543058796,4.1404274608,-3.8844257123
C	-1.4008922467,4.204800122,-6.9863053261
H	-2.1468879226,4.5610813574,-6.2735702746
H	-1.8476854283,3.4703784125,-7.6620027279
C	-0.2478586351,4.997566509,-3.5494980873
H	0.4808391763,5.0200053449,-4.3594937231
H	0.226149724,4.6651329562,-2.6262318964
C	0.2656598206,1.0591031266,-7.2646238532
C	-0.3512432704,0.542928773,-8.3704032426
S	1.9422396642,1.4292333826,-7.6271703265
C	0.4978954185,0.441860841,-9.5090209618
H	-1.3901168978,0.2418586668,-8.3672029141
C	1.7654335393,0.8762164189,-9.2632146023
H	0.1738571355,0.0624366775,-10.4683096294
H	2.6035958604,0.9143414642,-9.9406300902
C	-1.6467361648,2.1188527257,-1.8715871749
C	-1.1649267384,1.5283669887,-0.6999709751
S	-2.7053850167,3.4829942003,-1.5042491474
C	-1.6552569556,2.2275064026,0.4594640207
C	-2.4973657605,3.2598743014,0.1989689564
H	-0.6745668258,5.9884735392,-3.406005219
H	-1.0168729051,5.0414092589,-7.5671175513
H	-3.0061916108,3.9085642489,0.8955902611
H	-1.4102007497,1.9184285855,1.4678584279

TS for the CMD activation of the EHON naphthalene (C5) protons

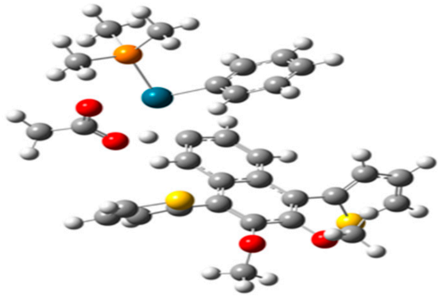

Pd -1.4146503401,0.4652339228,-0.3016585738
C -0.4106365102,4.4109801122,1.1341141887
C -0.8857267212,3.1032662484,1.0367348722
C -0.702304362,2.3646336788,-0.1329887715
C -0.0319531242,2.9621023495,-1.2021943752
C 0.4453188122,4.2695238381,-1.1045987934
C 0.254600764,5.0006441879,0.0634457944
O -2.2834707134,-1.5768812374,-0.6352549083
C -1.5032358005,-2.5548412481,-0.6078165827
P -3.3875161364,1.2976540211,-1.3239634008
O -0.2669306731,-2.4711366977,-0.2894727859
H -1.3882409407,2.6617005481,1.8891318613
H 0.9687294396,4.7131252164,-1.9445239224
H 0.6243347353,6.0161339925,0.1404177522
H -0.5582739829,4.9663595231,2.0538051207
H 0.1363938561,2.4106207565,-2.120738434
C -2.0112597687,-3.9340975107,-0.9517978648
H -1.972665512,-4.5607693011,-0.0586886289
H -3.0330030553,-3.8877269125,-1.3206402015
H -1.3596441953,-4.3912391233,-1.6970908981
H -0.0232164401,-1.3571844959,0.1071546046
C -3.4907900105,0.8725776579,-3.1199757193
H -4.4515316553,1.1721257988,-3.5441953125
H -2.6880136137,1.3760053573,-3.6589813245
H -3.3624519661,-0.2034840449,-3.2344240063
C -4.8994894517,0.5034075767,-0.620826946
H -5.7925627141,0.7812821457,-1.184476064
H -4.7646998138,-0.5772393322,-0.6446309112
H -5.0193001831,0.8123061488,0.4178858738
C -3.7912342163,3.0962955798,-1.2910049733
H -4.7378613999,3.2901517631,-1.7991634451
H -3.8567989427,3.4335047626,-0.2569412412
H -2.9923382218,3.657894343,-1.7736184298
C 0.4425563232,-0.1765119897,0.7960806746
C 0.2492836851,-0.277327371,2.1647703763
C 1.2499829695,0.0394355311,3.1175874086

C	2.5129951111,0.5160370888,2.6433346231
C	2.7226723036,0.5753687163,1.2410449323
C	1.7286347944,0.243380764,0.3598036094
C	1.0281270572,-0.1024403999,4.5271541149
C	3.5208151789,0.9105195389,3.5802884843
H	3.6887335151,0.8941455096,0.8744216011
C	3.2640412854,0.7860895303,4.9320463142
C	2.0282122748,0.2656816533,5.4039582932
O	4.2280872765,1.1173940154,5.8523117061
O	1.8254270129,0.1852114318,6.7608868744
C	4.034277168,2.3773073866,6.5113094429
H	3.0879135691,2.3917303303,7.0552757948
H	4.0639230363,3.1950553383,5.7862751561
C	2.4599591915,-0.9282772321,7.4059981341
H	3.5423694175,-0.8945273575,7.2686031566
H	2.0616662628,-1.8705399262,7.0200505884
C	4.8071267426,1.474375825,3.1217508561
C	5.0262325404,2.6520314529,2.4606609305
S	6.3209582361,0.6177796597,3.3488027228
C	6.3927087313,2.8830716562,2.1366595546
H	4.2250603097,3.3339996718,2.2100803154
C	7.210467557,1.8726077319,2.5452232098
H	6.746964742,3.765156843,1.6213275801
H	8.2797364232,1.7893105801,2.4328730378
C	-0.2389440659,-0.6568268228,5.0473678962
C	-0.7176646332,-1.9327302032,4.9307182695
S	-1.3889064753,0.3426057941,5.9170680692
C	-1.9930615993,-2.1256654673,5.5329572168
H	-0.1679387828,-2.7135587192,4.4228845035
C	-2.4868164928,-0.9894866146,6.0999723884
H	2.2201930978,-0.8422733378,8.464205566
H	4.8623438695,2.4842928312,7.2094460275
H	-3.4201297954,-0.8526900537,6.622592153
H	-2.5159608547,-3.0720796448,5.5423395224
H	1.9351679573,0.3026612693,-0.7031618846
H	-0.7029874823,-0.6263929056,2.5444062761

TS for the CMD activation of the EHON naphthalene (C6) protons

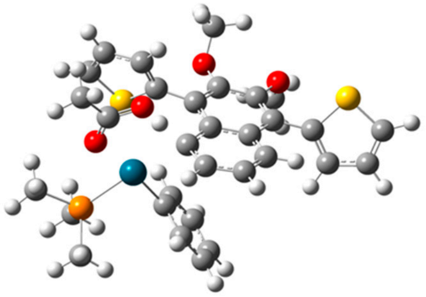

Pd -1.3473437793,0.2229254921,-0.215961302
C -0.3378645779,4.0475296479,1.561218076
C -0.8580042018,2.7725341743,1.3367243346
C -0.5980336177,2.0910226729,0.1466718897
C 0.1942466757,2.7213723638,-0.8164055402
C 0.713290584,3.9969163506,-0.5950737746
C 0.4478415451,4.6670380132,0.5948965897
O -2.224023693,-1.7471314247,-0.8138253525
C -1.6136533737,-2.8151666136,-0.6130040764
P -3.2483867353,1.3024198515,-1.1371087378
O -0.4733472454,-2.8925491939,-0.0295012899
H -1.4674570282,2.3157395169,2.1068252376
H 1.3306224678,4.4627964654,-1.3553547444
H 0.8508511766,5.6577796389,0.7679843721
H -0.5503629322,4.5542820063,2.4961958042
H 0.4285391137,2.2166561265,-1.7463413319
C -2.2169565912,-4.1261297496,-1.0544167472
H -2.5344074233,-4.6827784777,-0.1701754716
H -3.0763937506,-3.9546796895,-1.698019159
H -1.4682813635,-4.7282399395,-1.5692124953
H -0.1128560996,-1.8278653872,0.2922324219
C -2.9552902764,2.1954088636,-2.7277999
H -3.8783009045,2.6422677316,-3.1028816224
H -2.2121764808,2.9760708315,-2.5685332337
H -2.5679335925,1.4954548662,-3.4685245284
C -4.6169343779,0.1369842538,-1.5606842686
H -5.4558983708,0.6682171635,-2.0147113042
H -4.2411317095,-0.6234162701,-2.2429373638
H -4.9487950687,-0.3686104037,-0.6543766811
C -4.0979840817,2.5741476408,-0.1038460142
H -4.972539501,2.9721663348,-0.6223459532
H -4.4105782538,2.1223988493,0.8376490817
H -3.4040561363,3.3829315355,0.1194910574
C 1.5845869683,-0.4970296379,-0.3961401388
C 0.617955074,-0.5708958696,0.5975778796
C 1.0209812315,-0.257839875,1.943892472

C	2.353219915,0.2202770981,2.1921227203
C	3.2775216098,0.2637637816,1.1181858408
C	2.9108780498,-0.1104683336,-0.1475406764
C	0.1451686401,-0.4197900553,3.0691208891
C	2.7482720092,0.6427268835,3.503099698
H	4.2886170664,0.5901449086,1.317114034
H	3.6368095729,-0.0929568236,-0.9524827656
C	1.8543319981,0.5198037303,4.5442831739
C	0.5699565013,-0.0424121986,4.3308279875
O	2.2181142107,0.8808297339,5.8196462285
O	-0.2781816809,-0.1600853287,5.4061230903
C	1.666593166,2.12230868,6.2795701694
H	0.5753450588,2.0878908779,6.2833605657
H	2.013587568,2.9490655344,5.6538645714
C	0.0179857082,-1.237561655,6.3060424069
H	1.0205508267,-1.1342725731,6.7247503888
H	-0.0789350623,-2.1984090006,5.7936712511
C	4.0809684746,1.2335192098,3.7467841718
C	4.5631983515,2.4381104837,3.3139117367
S	5.311326552,0.3830668385,4.6631165107
C	5.9065130276,2.6944502667,3.7094343984
H	3.9675380232,3.121250885,2.7236491386
C	6.4463065401,1.6768446555,4.4371882392
H	6.4438726237,3.5992016922,3.4606381647
H	7.4352833132,1.6081345216,4.8615873364
C	-1.1842577681,-1.0683063656,2.9723701123
C	-1.4553082594,-2.4044500014,2.8947138881
S	-2.6836631227,-0.1631356716,3.1196440312
C	-2.8439542226,-2.7178421535,2.9349570463
H	-0.6756433343,-3.1470522011,2.8034805793
C	-3.6331658683,-1.6141069407,3.0507656469
H	-0.7219240665,-1.1783188474,7.1021613071
H	2.0341383877,2.2597410384,7.2948715486
H	-4.7089173782,-1.5611668735,3.1045910594
H	-3.2337103314,-3.7256028903,2.8876528798
H	1.3134670597,-0.763657205,-1.413622705

Photovoltaic characterization

Table S1. Photovoltaic characteristics for OPV devices.

**Device thickness (nm)**	V_{oc} **(V)**	J_{sc} **(mA·cm^-2^)**	***FF***	η **(%)**
26	0.70	0.66	0.19	0.11
53	0.80	0.58	0.20	0.12
64	0.80	0.51	0.18	0.09
72	0.75	0.39	0.18	0.07
97	0.75	0.05	0.19	0.01
110	0.65	0.03	0.20	0.01
